# Dalpiciclib plus chidamide in HR + /HER2−advanced breast cancer after CDK4/6 inhibitor failure: a phase Ib trial

**DOI:** 10.1038/s41467-026-70650-6

**Published:** 2026-03-20

**Authors:** Jinmei Zhou, Xuexue Wu, Yimeng Du, Jinyi Xiao, Xiaofeng Kang, Jiaxin Chen, Xiaobo Wang, Yanhong Tai, Li Bian, Shaohua Zhang, Zheng Pang, Yang Li, Zefei Jiang, Xiaojie Xu, Tao Wang

**Affiliations:** 1 Senior Department of Oncology, Chinese PLA General Hospital, Beijing, China; 2 Department of Genetic Engineering, Beijing Institute of Biotechnology, Beijing, China; 3 Department of Pathology, Chinese PLA General Hospital, Beijing, China; 4 Department of Medical Affairs, Jiangsu Hengrui Pharmaceuticals, Shanghai, China

**Keywords:** Breast cancer, Cancer therapeutic resistance

## Abstract

The optimal therapy after cyclin‑dependent kinase 4/6 inhibitor (CDK4/6i) failure in hormone receptor-positive/human epidermal growth factor receptor 2-negative (HR + /HER2 − ) advanced breast cancer (BC) remains undefined. In this study, we demonstrate that dalpiciclib combined with chidamide exerted synergistic antitumor effects in estrogen receptor-positive (ER + )/HER2 − BC cell lines and patient-derived organoids, providing a rationale for subsequent clinical evaluation. We conducte a single‑arm, phase Ib, Bayesian optimal interval dose‑escalation study (NCT05586841) evaluating dalpiciclib plus chidamide across four groups [A, 125 mg dalpiciclib mg/d and chidamide 25 mg twice a week (BIW); B, dalpiciclib 125 mg/d, chidamide 20 mg BIW; C, dalpiciclib 100 mg/d, 25 mg chidamide BIW; D, dalpiciclib 100 mg/d, chidamide 20 mg BIW]. The primary endpoint is maximum tolerated dose (MTD), and the secondary endpoints are objective response rate (ORR), progression‑free survival (PFS), disease control rate and safety. Among 22 enrolled patients, dose‑limiting toxicities occur in 3 patients, and the MTD is identified as group C. Grade 3–4 adverse events include neutropenia (100%), leukopenia (64%), and thrombocytopenia (36%). The ORR is 9.1% overall and 16.7% at the MTD, with median PFS of 5.8 months overall and 12.3 months at the MTD. Patients with PIK3CA mutations have shorter mPFS [5.04 months; 95% CI: 2.0−not estimable (NE)] compared to those with wild type (9.25 months; 95% CI: 1.97–NE). Here we show that dalpiciclib plus chidamide has manageable safety and preliminary antitumor activity in HR + /HER2− advanced BC following CDK4/6i failure.

## Abstract (web summary)

Hormone receptor-positive, HER2-negative advanced breast cancer lacks defined treatment options following CDK4/6 inhibitor (CDK4/6i) failure. Here, the authors present a phase Ib clinical trial where the CDK4/6i dalpiciclib is combined with the histone deacetylase inhibitor chidamide in this setting.

## Introduction

Breast cancer (BC) is a prevalent malignancy and the most serious threats to women’s health worldwide^1^. Among its various subtypes, hormone receptor-positive/human epidermal growth factor receptor 2-negative (HR + /HER2-) BC is the predominant molecular subtype, accounting for approximately 70% of all cases^2^. Despite advancements in clinical treatment have established that combining cyclin-dependent kinase 4/6 inhibitors (CDK4/6i) with endocrine therapy (ET) effectively improves patient prognosis, and this regimen has become the standard first-line treatment for advanced HR + /HER2- BC^3,4^. However, the acquired resistance significantly impacts patient outcomes, and there is no standard treatment after CDK4/6i treatment progression^5^. Current clinical approaches to overcoming endocrine resistance commonly involve switching to an alternative CDK4/6i or targeting other signaling pathways. Therefore, there is an urgent need to explore novel combination regimens to overcome CDK4/6i resistance.

Dalpiciclib is a novel emerged oral formulation CDK4/6i. The DAWNA-1 study demonstrated that dalpiciclib combined with fulvestrant significantly prolonged median progression-free survival (mPFS) compared to placebo plus fulvestrant [16.6 months (95% CI 15.2–18.6) vs. 7.2 months (95% CI 5.6–9.2), HR = 0.50] in patients with HR + /HER2- advanced BC who had recurrent or progressed after prior ET^6^. Chidamide, a selective histone deacetylase inhibitor (HDACi) that inhibits tumor cell proliferation and viability^7^. A multicenter, randomized, double-blind phase III clinical trial showed that the combination of chidamide and exemestane significantly improved PFS (7.4 months vs. 3.8 months)^8^. Previous studies have indicated that HDACi can induce G0/G1 cell cycle arrest, inhibiting the growth of multidrug-resistant BC cells^9^. Additionally, HDACi can suppress the transcription of *c-MYC*^10^, a gene associated with CDK4/6i resistance, suggesting a potential synergistic effect between the two agents. Although preclinical studies have demonstrated the potential synergistic antitumor effects of dalpiciclib and chidamide, clinical data have revealed similar toxicity profiles. These safety concerns underscore the need for optimized dosing strategies that balance efficacy with tolerability.

In this work, we evaluate the safety and preliminary efficacy of dalpiciclib plus chidamide in patients with HR + /HER2- advanced BC after CDK4/6i treatment failure. In this phase Ib, single-arm, dose-escalation trial, we identify the maximum tolerated dose (MTD) and observe that the combination shows manageable toxicity and antitumor activity, particularly in patients without *PIK3CA* mutations.

## Results

### Synergistic efficacy of dalpiciclib plus chidamide for BC treatment in vitro

**Fig. 1:**
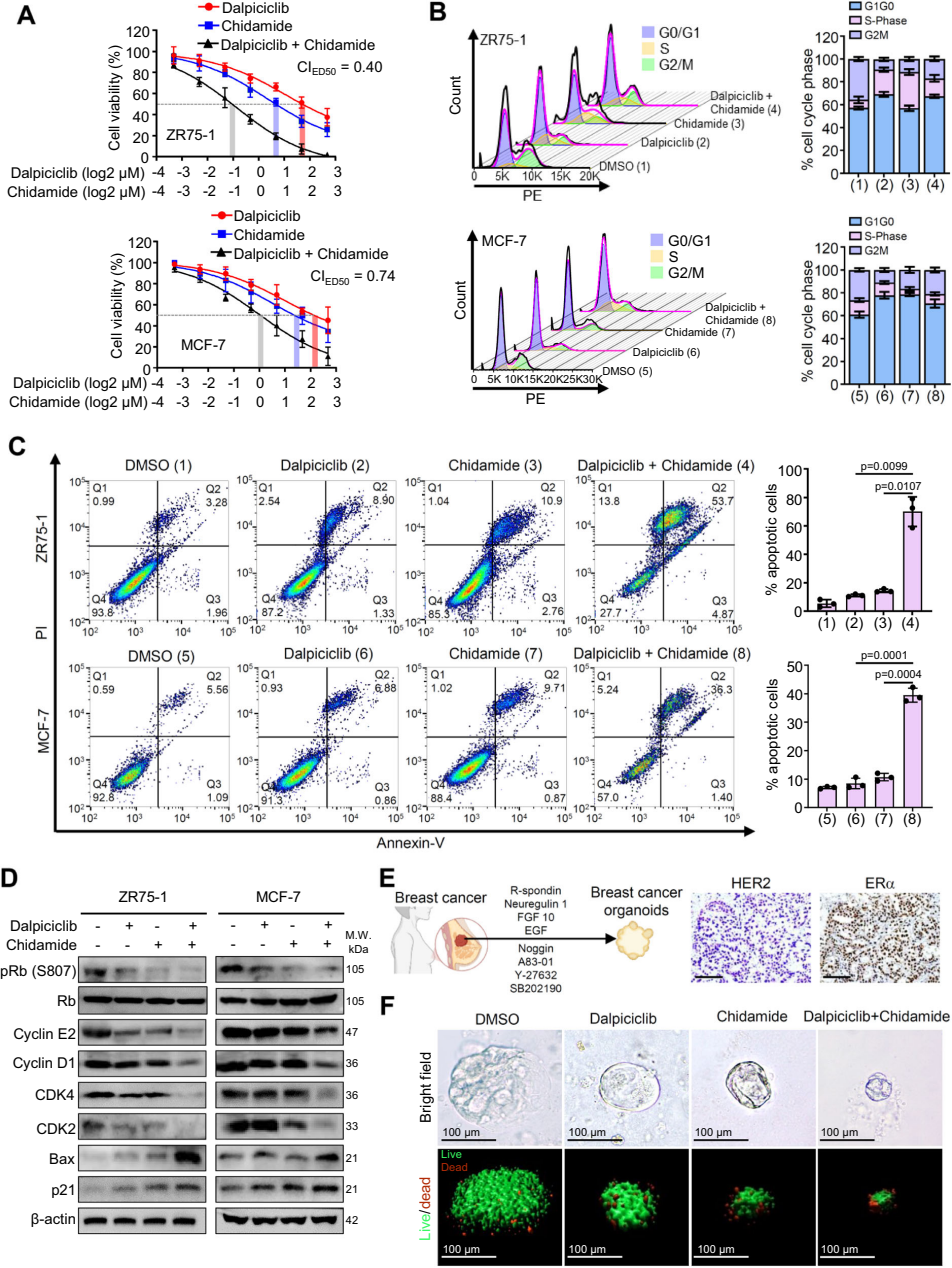
Validation of the synergistic effect of the combination of dalpiciclib and chidamide for BC treatment in vitro. **A** Cell viability assay in BC cells treated with indicated doses of dalpiciclib, chidamide, or the combination of dalpiciclib and chidamide. Cell viability was normalized to the untreated controls. Data are pooled from three independent experiments. **B** Cell cycle analysis of BC cells treated with dalpiciclib (2 μM), chidamide (2 μM), or their combination. Right panels showing the percentage of each cell cycle phase. Data are pooled from three independent experiments. **C** Apoptosis assay of BC cells treated as in (**B**). Apoptosis was assessed using flow cytometry. Right panels showing the percentage of apoptotic cells. Data are pooled from three independent experiments, and statistical significances were assessed using student’s *t* test. **D** Western blot analysis of key regulators of cell cycle and apoptosis modulated by CDK4/6i in BC cells. The blot is representative of three independent experiments. **E** Schematic diagram illustrating the establishment and culture BC organoids. Scale bar = 50 μm. This diagram was created in BioRender. Du, Y. (2025) https://BioRender.com/463stvc. **F** BC organoids treated as in (**B**) and subjected to live/dead staining. The representative bright field and fluorescent images of three independent experiments showing the size and live/dead staining of organoids. Scale bar = 100 μm. Data represent the mean ± SD. Source data are provided as a Source Data file.

The antitumor effect of dalpiciclib plus chidamide was validated in two ER + /HER2- BC cell lines with different *PIK3CA* status, ZR75-1 (*PIK3CA*-wildtype) and MCF-7 (*PIK3CA*-mutant). In both BC cells, the combination therapy significantly enhanced the cytotoxicity compared to dalpiciclib or chidamide monotherapy, as evidenced by the combination index (CI) at median effect dose (ED50) less than 1 (Fig. 1A). Flow cytometry analysis further demonstrated that, compared with monotherapy, combination therapy resulted in a marked increase in the proportion of cells in G0/G1 phase and G2/M phase as well as apoptotic cells, but a reduction in the proportion of cells in S-phase (Fig. 1B, C). The gating strategy used for FACS analysis is illustrated in Supplementary Fig. S3. Western blot analysis revealed that the combination treatment markedly suppressed key cell cycle regulators, including phosphorylated Rb (pRb), cyclin E2, cyclin D1, CDK4, and CDK2, while upregulating pro-apoptotic proteins Bax and p21 (Fig. 1D). It should be noted that this synergistic effect was relatively weak in MCF-7 cells compared to ZR75-1 cells, indicating that *PIK3CA*-wildtype cells are more sensitive to combination therapy of dalpiciclib and chidamide.

The combination efficacy was further evaluated in BC organoids from the ER + /HER2- patients (Fig. 1E). Either treatment of dalpiciclib or chidamide alone effectively reduced the organoid size and viability. Importantly, in comparison with monotherapy, the organoids treated by dalpiciclib plus chidamide resulted in a more substantial shrinkage and a higher proportion of dead cells (Fig. 1F). These results collectively suggest that combination of dalpiciclib plus chidamide exerts a synergistic antitumor effect in BC in vitro.

### Patients

**Fig. 2:**
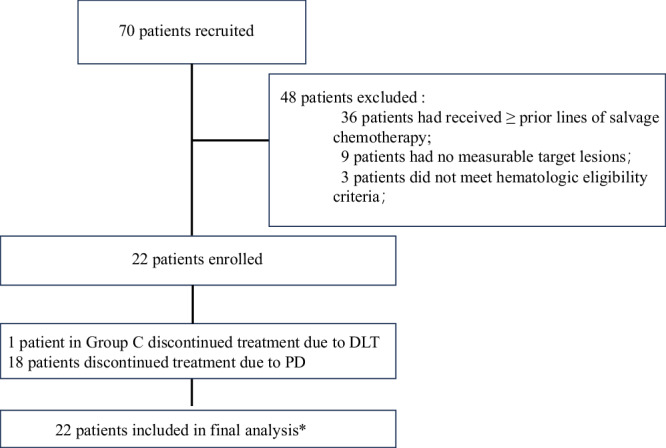
Patient flowchart. * 3 patients in Group C were still on treatment at the time of analysis, DLT dose-limiting toxicity, PD progressive disease.

From January 2023 to May 2024, a total of 70 patients with HR + /HER2 − BC who had experienced disease progression on CDK4/6i were screened for enrollment. Of these patients, 36 had received > 2 prior lines of salvage chemotherapy, 9 lacked measurable target lesions, and 3 did not meet hematologic criteria. A total of 22 patients were enrolled into four cohorts according to the Bayesian optimal interval (BOIN) design: 4 in Group A, 3 in Group B, 12 in Group C, and 3 in Group D. One Group C patient discontinued due to a dose-limiting toxicity (DLT), three remained on treatment at data cutoff, and the rest discontinued due to progressive disease. All patients were included in the final analysis. (Fig. 2). The median age of 51.5 years (range: 33–63). Most patients (95.5%, 21/22) had an Eastern Cooperative Oncology Group (ECOG) performance status of 0, while only one patient (4.5%) had an ECOG score of 1. In terms of HR status, 63.6% (14/22) of patients were estrogen receptor-positive/progesterone receptor-positive (ER + / PR + ), and 36.4% (8/22) were ER + /PR − . The majority of patients (59.1%, 13/22) had ≥ 3 metastatic sites, with visceral metastases (77.3%, 17/22) and bone metastases (72.7%, 16/22) being the most common. Lymph node metastases were observed in 59.1% (13/22) of patients. Regarding prior treatments, 81.8% (18/22) of patients had received chemotherapy for recurrent/metastatic disease, and 54.5% (12/22) had received ≥ 3 lines of prior therapy. Among CDK4/6i in prior treatment, abemaciclib was the most frequently used (81.8%, 18/22), followed by palbociclib (40.9%, 9/22) and ribociclib (4.5%, 1/22). The majority of patients (68.2%, 15/22) had received CDK4/6i for more than 12 months (Table 1).

**Table 1:** The baseline characteristics of patients

Demographics	A (N = 4)	B (N = 3)	C (N = 12)	D (N = 3)	ALL (N = 22)
Age (years)					
Median age, (range)	51 (39–59)	43 (33–54)	50.5 (36–61)	59 (52–63)	51.5 (33–63)
ECOG, *n* (%)					
0	4 (100%)	3 (100%)	11 (91.7%)	3 (100%)	21 (95.5%)
1	0	0	1 (8.3%)	0	1 (4.5%)
2	0	0	0	0	
HR status, *n* (%)					
ER + /PR+	3 (75%)	2 (66.7%)	7 (58.3%)	2 (66.7%)	14 (63.6%)
ER + /PR-	1 (25%)	1 (33.3%)	5 (41.7%)	1 (33.3%)	8 (36.4%)
ER-/PR+	0	0	0	0	0
Number of metastatic sites, *n* (%)					
< 3	2 (50%)	0	7 (58.3%)	0	9 (40.9%)
≥ 3	2 (50%)	3 (100%)	5 (41.7%)	3 (100%)	13(59.1%)
Metastatic site, *n* (%)					
visceral metastasis	3 (75%)	2 (66.7%)	9 (75%)	3 (100%)	17 (77.3%)
bone metastasis	3 (75%)	2 (66.7%)	8 (66.7%)	3 (100%)	16 (72.7%)
lymph node metastasis	2 (50%)	3 (100%)	6 (50%)	2 (66.7%)	13 (59.1%)
Previous lines of therapy*, *n* (%)					
1	0	0	1 (8.3%)	1 (33.3%)	2 (9.1%)
2	1 (25%)	1 (33.3%)	5 (41.7%)	1 (33.3%)	8 (36.4%)
≥ 3	3 (75%)	2 (66.7%)	6 (50%)	1 (33.3%)	12 (54.5%)
Previous chemotherapy for recurrent/metastatic disease, *n* (%)					
yes	4 (100%)	3 (100%)	10 (83.3%)	1 (33.3%)	18 (81.8%)
no	0	0	2 (16.7%)	2 (66.7%)	4 (18.2%)
Previous CDK4/6i for recurrent/metastatic disease, *n* (%)					
Palbociclib	1 (25%)	2 (66.7%)	5 (41.7%)	1 (33.3%)	9 (40.9%)
Ribociclib	0	0	1 (8.3%)	0	1 (4.5%)
Abemaciclib	4 (100%)	2 (66.7%)	9 (75%)	3 (100%)	18 (81.8%)
Duration of Previous CDK4/6i, *n* (%)					
≤ 12 m	2 (50%)	1 (33.3%)	2 (16.7%)	2 (66.7%)	7 (31.8%)
> 12 m	2 (50%)	2 (66.7%)	10 (83.3%)	1 (33.3%)	15 (68.2%)
Gene Alteration	A (*N* = 1)	B (*N* = 2)	C (*N* = 8)	D (*N* = 3)	ALL (*N* = 14)
*PIK3CA* mutation	0	2 (100%)	4 (50%)	2 (66.7%)	8 (57.1%)
*PTEN* mutation	0	0	1 (12.5%)	0	1 (7.1%)
*ESR1* mutation	1 (100%)	0	2 (25%)	0	4 (28.6%)
*CCND1, FGF3, FGF4* and *FGF19* amplification	1 (100%)	0	4 (50%)	0	5 (35.7%)
*TP53*	0	1 (50%)	3 (37.5%)	0	4 (28.6%)

*CDK 4/6i* cyclin-dependent kinase 4/6 inhibitors, *ER* estrogen receptor-negative, *PR* progesterone receptor-positive.

### Dose escalation

Dose escalation followed a Bayesian optimal interval (BOIN) design. The first cohort [Group D: dalpiciclib 100 mg/day plus chidamide 20 mg twice a week (BIW)] enrolled three patients, with no DLTs observed, allowing escalation to Group B (dalpiciclib 125 mg/d, chidamide 20 mg BIW), where again no DLTs were reported. The next dose level, Group C (dalpiciclib 100 mg/d, 25 mg chidamide BIW), enrolled four patients, among whom one experienced a DLT (25%). According to the BOIN design, the dose level was escalated to Group A (125 mg dalpiciclib daily and chidamide 25 mg BIW). In Group A, one of four patients (25%) experienced a DLT, below the predefined escalation threshold of 26% (Fig. S1).

Across all cohorts, three patients (13.6%) experienced DLTs—all grade 4 thrombocytopenia occurring within the first treatment cycle. DLTs were observed in Group A (1/4, 25.0%; 95% CI: 0.63–80.59) and Group C (2/12, 16.7%; 95% CI: 2.09–48.41), with no DLTs in Groups B or D (0/3 each; 95% CI: 0–70.76) (Table 2).

**Table 2:** Dose-limiting toxicities analysis

Events	A (N = 4)	B (N = 3)	C (N = 12)		D (N = 3)
DLT, n (%, 95%CI)	1 (25%, 0.63–80.59)	0 (0, 0–70.76)	2 (16.7%, 2.09–48.41)	0 (0, 0–70.76)	

*DLT* dose limit toxicity.

Although the Group A DLT rate was below the BOIN escalation threshold, all four patients experienced treatment interruptions due to adverse events within the first two cycles. Considering both the safety profile and preliminary efficacy, the dose was de-escalated to Group C, which ultimately enrolled 12 patients without further de-escalation per protocol.

While all patients in Group C eventually required chidamide dose reductions, these occurred outside the DLT observation window. Based on protocol-defined rules and overall assessment, the regimen in Group C was selected as the MTD.

### Safety

All 22 patients (100%) experienced at least one adverse event (AE) of any grade, and all (100%) also developed grade 3–4 AEs. Grade 4 AEs were reported in 54.5% (12/22) of patients, with varying incidence across dose groups: two patients in Group A, 1 in Group B, 8 patients in Group C, and 1 in Group D. No treatment-related deaths were observed during the study period. The common grade 3–4 AEs were neutropenia (100%), leukopenia (64%) and thrombocytopenia (36%) (Table S3). Dalpiciclib dose reductions were required in 31.8% of patients, occurring in all patients (7/22) in Groups A (*n* = 4) and B (*n* = 3) but not in Groups C or D. In contrast, chidamide dose reductions were more frequent, required in 72.7% (16/22) of patients, including all patients in Groups A (*n* = 4) and C (*n* = 12), while no reductions were observed in Groups B and D (Table 3).

**Table 3:** Adverse Events

Events	A (*N* = 4)	B (*N* = 3)	C (*N* = 12)	D (*N* = 3)	ALL (*N* = 22)
Any grade AE	4 (100%)	3 (100%)	12 (100%)	3 (100%)	22 (100%)
Grade 3–4 AE	4 (100%)	3 (100%)	12 (100%)	3 (100%)	22 (100%)
Grade 4 AE	2 (50%)	1 (33.3%)	8 (66.7%)	1 (33.3%)	12 (54.5%)
Led to death	0	0	0	0	0
AE induced dose reduction, *n* (%)					
Dalpiciclib	4 (100%)	3 (100%)	0	0	7 (31.8%)
Chidamide	4 (100%)	0	12 (100%)	0	16 (72.7%)

*AE* adverse events.

### Efficacy

**Fig. 3:**
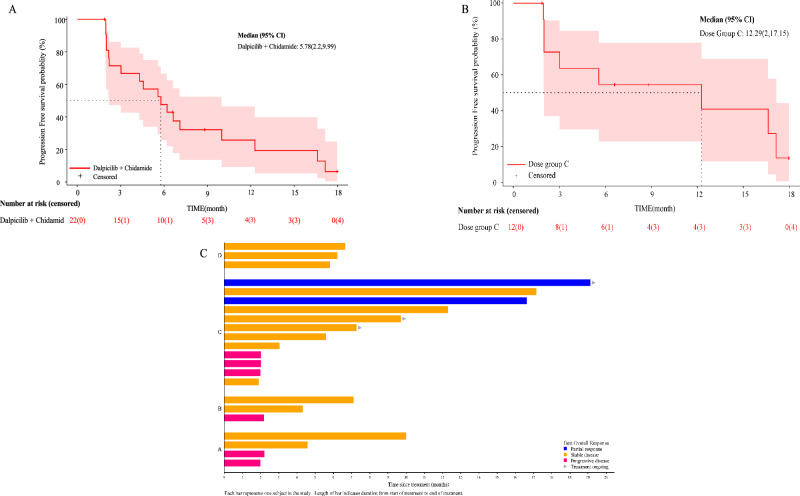
Clinical efficacy of dalpiciclib plus chidamide in patients with HR+/HER2− advanced BC after CDK4/6i failure. **A** PFS of all patients and **B** PFS of group C, **C** Swimmer plot depicting treatment duration and best overall response for individual patients across different dose groups. Each bar represents a single patient. Source data are provided as a Source Data file.

**Fig. 4:**
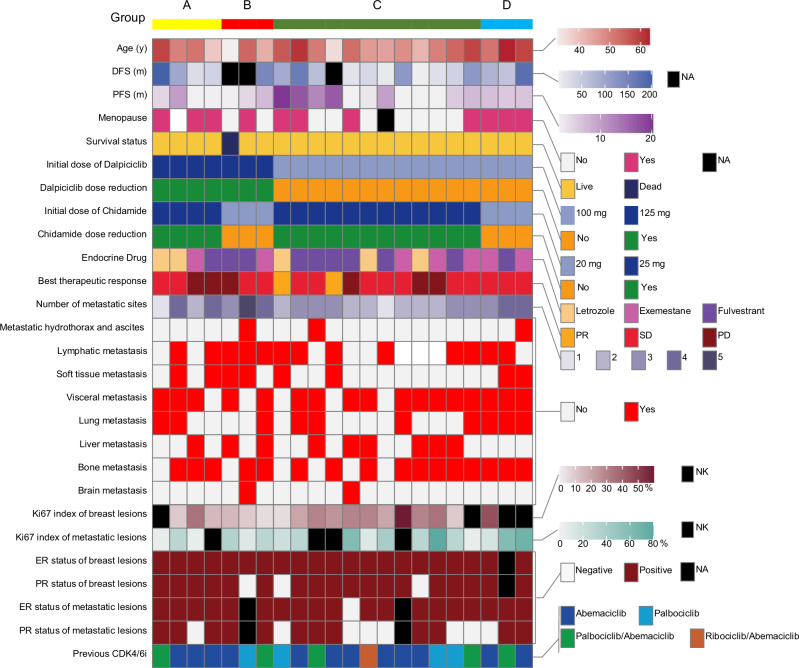
Baseline characteristics, treatment details, and therapeutic responses of enrolled patients. Each column represents an individual patient. Panels A–D correspond to Groups A–D, respectively. Source data are provided as a Source Data file.

As shown in Table 4, the ORR was 9.1% (2/22, 95% CI: 1.1–29.2%), with both partial responses (PRs) observed in Group C (16.7%, 2/12, 95% CI: 2.1–48.4%). No complete responses (CRs) were observed. The disease control rate (DCR) was 68.2% (15/22), with the highest DCR observed in Group D (100%, 3/3), followed by Group C (66.7%, 8/12), Group B (66.7%, 2/3), and Group A (50.0%, 2/2). Stable disease (SD) was the best response in 63.6% (14/22) of patients, while 27.3% (6/22) experienced progressive disease (PD). The proportion of patients with PD was 50.0% (2/2) in Group A, 33.3% (1/3) in Group B, 25.0% (3/12) in Group C, and 0% (0/3) in Group D. The mPFS of all eligible patients was 5.78 months (95% CI: 2.2–10.0) (Fig. 3A) and the mPFS of Group C was 12.29 months (95% CI: 2.0–17.2) (Fig. 3B). The treatment duration and response of individual patients were depicted in Fig. 3C. Baseline clinicopathological characteristics, treatment details, and individual responses for all patients are summarized in Fig. 4.

**Table 4:** Efficacy

Endpoints	A (N = 4)	B (N = 3)	C (N = 12)	D (N = 3)	ALL (N = 22)
Best treatment response, *n* (%)					
CR	0	0	0	0	0
PR	0	0	2 (16.7%)	0	2 (9.1%)
SD	2 (50%)	2 (66.7%)	7 (58.3%)	3 (100%)	14 (63.6%)
PD	2 (50%)	1 (33.3%)	3 (25%)	0	6 (27.3%)
ORR, *n* (%)	0	0	2 (16.7%)	0	2 (9.1%)
DCR, *n* (%)	2 (50%)	2 (66.7%)	8 (66.7%)	3 (100%)	15 (68.2%)

*ORR* objective response rate, *DCR* disease control rate, *CR* complete response, *PR* partial response, *SD* stable disease, *PD* progressive disease.

### Subgroup analysis

**Fig. 5:**
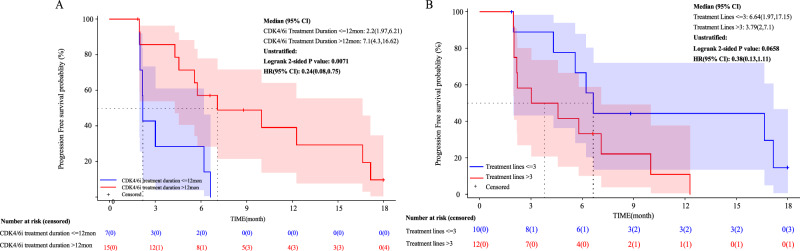
Subgroup analysis of PFS according to prior treatment characteristics. Kaplan–Meier curve of PFS stratified by. **A** prior CDK4/6i treatment duration and **B** number of prior therapy lines. Source data are provided as a Source Data file.

Subgroup analyses were not prespecified in the study protocol and were conducted descriptively. In the subgroup analysis of PFS, patients were stratified based on the duration of prior CDK4/6i treatment and the number of prior therapy lines. Patients with a prior CDK4/6 inhibitor treatment duration of more than 12 months had a median PFS of 7.1 months (95% CI: 4.3–16.62), whereas those with a duration of ≤ 12 months had a median PFS of 2.2 months (95% CI: 1.97–6.21) (HR = 0.24, 95% CI: 0.08–0.75, *p* = 0.0071) (Fig. 5A). Regarding prior therapy lines, patients who had received ≤ 3 lines of therapy exhibited a mPFS of 6.64 months (95% CI: 1.97–17.15), while those with > 3 prior therapy lines had a mPFS of 3.79 months (95% CI: 2.7–7.1) (HR = 0.38, 95% CI: 0.13–1.1, *p* = 0.0658) (Fig. 5B).

### Biomarker Analysis

**Fig. 6:**
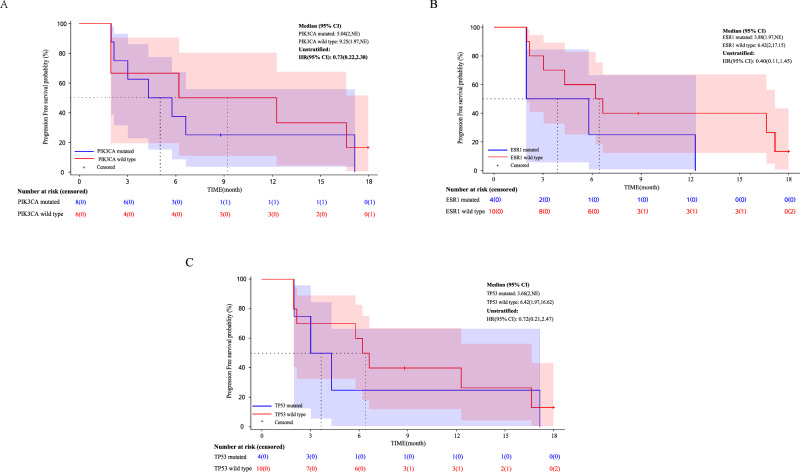
Association between genomic alterations and PFS. Kaplan–Meier curve of PFS for patients stratified by. **A** *PIK3CA*, **B** *ESR1* and **C** *TP53* mutation. Source data are provided as a Source Data file.

Biomarker analyses were not prespecified in the approved study protocol and were performed descriptively. Genetic profiling was performed in 14 of the 22 enrolled patients. Among those tested, *PIK3CA* mutations were identified in 57.1% (8/14), *ESR1* mutations in 28.6% (4/14), and amplifications in *CCND1*, *FGF3*, *FGF4*, and *FGF19* in 35.7% (5/14). *TP5*3 mutations were found in 28.6% (4/14), while *PTEN* mutations were observed in 7.1% (1/14). Genetic data were not available for the remaining 8 patients, who did not undergo molecular testing. These details are summarized in the Table 1. In the biomarker analysis, PFS was evaluated based on genetic mutation status. Patients with *PIK3CA* mutations had a mPFS of 5.04 months [95% CI: 2.0–not estimable (NE)], whereas those without *PIK3CA* mutations exhibited a mPFS of 9.25 months (95% CI: 1.97–NE). The HR for PFS was 0.73 (95% CI: 0.22–2.38) (Fig. 6A). Additionally, the analysis of *ESR1* and *TP53* mutations revealed variations in PFS. Patients with *ESR1* mutations had a mPFS of 3.88 months (95% CI: 2.0–NE), whereas those without *ESR1* mutations had a mPFS of 6.42 months (95% CI: 2.0–17.2), with a HR of 0.4 (95% CI: 0.1–1.5) (Fig. 6B). Similarly, for *TP53* mutations, the mPFS was 3.66 months (95% CI: 2.0–NE) in the mutation-positive group, compared to 6.42 months (95% CI: 2.0–16.6) in the wild-type group, with an HR of 0.7 (95% CI: 0.2–2.5) (Fig. 6C).

## Discussion

This study evaluated the dose escalation, efficacy, and safety of dalpiciclib in combination with chidamide in patients with HR + /HER2- BC who had progressed on prior CDK4/6i therapy. The MTD was established at Group C (dalpiciclib 100 mg/day, chidamide 25 mg twice weekly). The results demonstrated that this combination was tolerable and exhibited preliminary antitumor activity.

The MTD was determined as dalpiciclib 100 mg/d plus chidamide 25 mg twice weekly, based on the predefined DLT criteria. Three patients experienced DLTs, all of which were grade 4 thrombocytopenia, a known toxicity associated with chidamide and other histone deacetylase inhibitors^8,11^. Hematologic toxicities frequently observed with chidamide may result from its inhibition of rapidly proliferating hematopoietic stem and progenitor cells^12,13^. Similarly, dalpiciclib as a CDK4/6i that disrupts cell cycle progression, dalpiciclib can cause bone marrow suppression, contributing to neutropenia and thrombocytopenia. The observed DLTs were also consistent with the hematologic toxicities reported in prior studies of dalpiciclib^14^. These events were generally manageable with appropriate supportive care and dose modifications. No treatment-related deaths occurred. Overall, the tolerability of the current combination regimen is acceptable.

The mPFS was 5.8 months in all patients, with Group C showing a mPFS of 12.3 month. Our results appear to be favorable when compared to previously reported studies that evaluated treatment strategies after progression with CDK4/6i. In the MAINTAIN study, continuation of ribociclib plus ET after progression on a prior CDK4/6i resulted in mPFS of 5.3 months versus 2.7 months with placebo plus ET (HR: 0.57, 95% CI: 0.39–0.95, *p* = 0.006)^15^. Moreover, the postMONARCH trial revealed that abemaciclib plus fulvestrant group significantly prolonged mPFS [6.0 (95% CI, 5.6–8.6) versus 5.3 (95% CI, 3.7–5.6) months] compared to placebo combined fulvestrant arm^16^.In the PACE trial, patients receiving palbociclib plus fulvestrant achieved mPFS of 4.6 months, while the addition of avelumab increased mPFS to 8.1 months^17^. In the PALMIRA trial, mPFS was 4.2 months (95% CI, 3.5–5.8) in the palbociclib plus ET group versus 3.6 months (95% CI, 2.7–4.2) in the ET alone group (HR = 0.8, 95% CI, 0.6–1.1; *p* = 0.206)^18^. The EMBER-3 trial revealed a mPFS of 9.1 months in the CDK4/6i-pretreated subgroup^19^, However, this is not the only study reporting extended PFS in this setting. The phaseII ELAINE‑2 trial, evaluating lasofoxifene in combination with abemaciclib, also achieved a median PFS exceeding 12months in patients previously treated with CDK4/6i and ET^20^. Nevertheless, differences in patient populations across studies must be considered. In PACE, patients had received ≤ 2 prior lines of ET and 0–1 lines of chemotherapy in the advanced BC^17^. PALMIRA and postMONARCH trials enrolled patients who had received only one prior line of palbociclib or abemaciclib plus ET, and no other prior treatment^16,18^. Similarly, EMBER-3 trial enrolled patients with prior CDK4/6i plus ET with no other previous therapy^19^. The MAINTAIN trial enrolled patients who had 0–1 lines of chemotherapy and progressed on standard first-line treatment^15^. In contrast, patients in our study had received ≥ 2 prior lines of ET, representing a more heavily pretreated population. Nevertheless, the overall mPFS observed in this study was comparable to that reported in previous CDK4/6i studies. Notably, the mPFS achieved in Group C exceeded the results of previous studies. Although further validation is required, these results highlight the potential of dalpiciclib in combination with chidamide as a therapeutic strategy for advanced BC patients with endocrine-resistant disease following CDK4/6i therapy.

In the present study, mPFS was notably longer in Cohort C (12.3 months) compared to the overall population (ALL group, 5.78 months). Several factors may account for this observed difference. First, the relatively small sample size may have contributed to the observed differences. Second, the proportion of patients with more than three metastatic lesions was lower in Cohort C, suggesting a relatively lower disease burden compared to the ALL group and particularly to other cohorts (A, B, and D), where a higher proportion of patients presented with > 3 metastases. Third, patients in Cohort C had a longer duration of prior CDK4/6i therapy, with 65% of patients receiving treatment for more than 12 months, and a longer average duration of prior CDK4/6i treatment (17.5 months vs. 16.2 months in the ALL group). This may indicate a more favorable response to CDK4/6 inhibition in this subgroup, potentially contributing to the extended PFS observed with subsequent dalpiciclib-based combination therapy.

The safety profile observed in this study was consistent with previously reported toxicities of dalpiciclib and chidamide. The most common grade 3/4 AEs were neutropenia (100%), leukopenia (64%), and thrombocytopenia (36%). These toxicities are similar to those reported in clinical trials of other CDK4/6i, such as ribociclib and palbociclib^14,15^. No new safety signals were observed, and the incidence of grade 3/4 AEs was comparable to historical data for both agents. Overall, the combination therapy was well-tolerated and had an acceptable safety profile, making it a viable option for patients with HR + /HER2- BC who have progressed on prior CDK4/6i treatment.

Biomarker analysis provided insights into the differential treatment response among molecular subgroups. Patients with *PIK3CA* mutations had a mPFS of 5.04 months (95% CI: 2.0–NE), whereas those without mutations had mPFS of 9.25 months (95% CI: 1.97–NE) (HR: 0.73, 95% CI: 0.22–2.38). These findings are consistent with previous reports that *PIK3CA* mutations are associated with resistance to ET and CDK4/6i^21,22^. Notably, patients with *PIK3CA*-mutant HR + /HER2- BC derive greater benefit from PI3K inhibitors such as inavolisib, which was shown in the INOVA120 trial to improve mPFS to 15.0 months when combined with palbociclib-fulvestrant, compared to 7.3 months with placebo plus palbociclib-fulvestrant (HR: 0.43, 95% CI: 0.32–0.59; *P* < 0.001)^23^. Similarly, *ESR1* mutations were associated with reduced PFS (3.88 months vs. 6.42 months, HR: 0.4, 95% CI: 0.1–1.5) in our study. While *ESR1* mutations are well established as a mechanism of resistance to ET, their impact on the efficacy of CDK4/6i remains uncertain^24,25^, Most clinical studies to date have not demonstrated a significant difference in CDK4/6i benefit between tumors with and without *ESR1* mutations, suggesting that CDK4/6 inhibition may retain activity in this setting^26,27^. However, preclinical findings are limited and somewhat conflicting, one study indicated preserved CDK4/6i sensitivity in *ESR1*-mutant models^28^, whereas another associated specific hotspot mutations, such as Y537S and D538G, with resistance to CDK4/6i^29^. These discrepancies highlight the need for further investigation to clarify the clinical relevance of *ESR1* mutation status in this context. For *TP53* mutations, mPFS was 3.66 months in mutation-positive patients compared to 6.42 months in wild-type patients (HR: 0.7, 95% CI: 0.2–2.5), in line with prior studies suggesting that *TP53* alterations are associated with more aggressive tumor biology and reduced treatment efficacy^30^.

Our in vitro assay data demonstrate that in ER + /HER2− cultured BC models, dalpiciclib combined with chidamide exhibits enhanced cytotoxicity compared to monotherapy. This effect is supported by an increased proportion of cells arrested in the G0/G1 and G2/M phases and elevated levels of apoptosis, suggesting a synergistic interaction between dalpiciclib and chidamide. Mechanistically, this synergy is underpinned by the downregulation of critical cell cycle regulators (pRb, cyclin D1/E2, CDK4/2) and upregulation of pro-apoptotic markers (Bax, p21), consistent with previous findings on the molecular effects of HDACi and CDK4/6i^31–34^. Notably, ZR75-1 cells (PIK3CA wild-type) were more sensitive to combination treatment than MCF-7 cells (*PIK3CA* mutant), suggesting that *PIK3CA* mutational status may influence therapeutic response. This observation aligns with our prior work implicating *PIK3CA* mutations in resistance to the HDACi tucidinostat^35^. Furthermore, the antitumor efficacy of the combination regimen was validated in patient-derived BC organoids, which showed more pronounced shrinkage and cell death following treatment. These findings provide strong preclinical rationale for further clinical investigation of the dalpiciclib and chidamide combination in molecularly defined HR + /HER2 − BC populations.

There are several limitations to this study. First, the study was conducted at a single center with a relatively small sample size, which may limit the generalizability of the findings. Second, the observed differences in efficacy between Group C and the overall patient population may be attributable to the small sample size, as well as the heterogeneity of the patient population. Third, the lack of pharmacokinetic data is another limitation of the study, as understanding the pharmacokinetic profile of dalpiciclib in combination with chidamide could help optimize dosing strategies and improve patient outcomes.

In conclusion, this study establishes dalpiciclib in combination with chidamide as a feasible and tolerable treatment strategy for HR + /HER2- BC patients who have progressed on prior CDK4/6i.

## Methods

### Study design

This study is an open-label, single-arm, dose-escalation phase Ib clinical trial (NCT05586841) conducted between January 2023 and May 2024 at the Fifth Medical Center of the Chinese PLA General Hospital. This study was designed and conducted as an investigator-initiated trial (IIT). The study was conducted in compliance with the Declaration of Helsinki and Good Clinical Practice (GCP) guidelines. The protocol and its amendments approved by Ethics Committee of Hainan Jialong Internet Hospital. Informed consent was obtained from each patient prior to treatment.

### Patient eligibility

The key inclusion criteria were as follows: age ≥ 18 years old; ER+ and/or PR+ tumors (≥ 10%) and HER2- local recurrent or metastatic disease diagnosed by pathohistological or cytological examination; at least one extracranial measured lesion per Response Evaluation Criteria in Solid Tumors (RECIST, version 1.1); received ≤ 1 line of chemotherapy for recurrent or metastatic BC; experienced disease recurrence and/or metastasis after treatment with a CDK4/6i (palbociclib, abemaciclib, or ribociclib); received ≤ 3 lines of ET for recurrent or metastatic BC; ECOG performance status of 0–2; life expectancy of ≥ 6 months; normal hematologic, cardiac, hepatic, renal, and thyroid function. Patients previously received HDACi therapy; prior treatment with dalpiciclib; magnetic resonance imaging (MRI) or lumbar puncture-confirmed leptomeningeal metastasis, or imaging-confirmed central nervous system metastasis were excluded from this study. No sex- or gender-specific subgroup analyses were conducted. Dalpiciclib and chidamide were provided free of charge as study drugs as compensation for participation. Concomitant endocrine therapy was administered using agents included in the National Reimbursement Drug List and was reimbursed according to national health insurance policies, with any remaining costs covered by the patients. No alternative treatments were provided free of charge by the hospital.

### Intervention

This clinical trial employed the BOIN design^36^ to determine the MTD. Four dose groups were established as follows: Group A (125 mg dalpiciclib daily and chidamide 25 mg BIW), Group B (dalpiciclib 125 mg/d, chidamide 20 mg BIW), Group C (dalpiciclib 100 mg/d, 25 mg chidamide BIW); and Group D (dalpiciclib 100 mg/d, chidamide 20 mg BIW).

DLTs were defined in the protocol as treatment-related adverse events (TRAE) occurring within the first treatment cycle based on the Common Terminology Criteria for Adverse Events (CTCAE, Version 5.0) criteria (Table S1). Group D (dalpiciclib 100 mg/d, chidamide 20 mg BIW) was the initial dose and the dose-escalation scheme was Group D, Group B (dalpiciclib 125 mg/d, chidamide 20 mg BIW), Group C (dalpiciclib 100 mg/d, 25 mg chidamide BIW) and Group A (125 mg/d dalpiciclib and chidamide 25 mg BIW). The principles of dose titration were detailed in Table S2 and Fig. S2. The ET regimen was determined based on the clinical practice. Patients were treated according to the study protocol until disease progression or unacceptable toxicity.

### Assessment and endpoint

The primary endpoint was MTD, and the secondary endpoints were objective response rate (ORR), PFS, DCR and safety analysis. Eligible patients underwent weekly (± 2 days) routine blood tests, as well as liver and renal function assessments after drug administration. Tumor response was evaluated every two cycles (± 7 days) using imaging based on RECIST 1.1 criteria, until disease progression or the initiation of a new anti-tumor therapy. Patients who experienced disease progression or started a new treatment were followed up every 12 weeks to assess survival status.

The assessment of safety included AEs, clinical laboratory examinations, vital signs, and physical examinations. The AEs were documented and graded according to the CTCAE (version 5.0). Furthermore, we also explored the potential biomarkers associated with outcomes.

### Biomarker analysis

Tissue samples were obtained from FFPE blocks and processed for DNA extraction using the C1102-Concertbio kit, while blood samples were extracted using the C1002-Concertbio kit. DNA concentration and purity were assessed with a NanoDrop spectrophotometer. Targeted sequencing was performed using the 1123-gene panel (ChosenMed) with capture-based enrichment. Libraries were prepared with the KAPA Hyper Prep Kit, hybridized, and sequenced on an MGISEQ2000 platform (paired-end 100 bp reads). Raw data were processed using Fastp for quality control and aligned to the hg19 reference genome with BWA. Somatic variants were detected using GATK (Mutect2), VarDict, and VarScan, with an LoD of 1% mutant frequency. Germline variants were called with GATK, with an LoD of 15%. Annotation was performed using ANNOVAR, VEP, and SnpEff, incorporating databases such as dbSNP, ClinVar, and COSMIC. Filtering criteria excluded intronic, common, synonymous, and low-support variants.

### Cell viability assay

For cell viability assays, MCF-7 or ZR75-1 cells were seeded into 96-well plates, and dalpiciclib, chidamide or combination of dalpiciclib and chidamide at indicated concentrations were added for 72 h treatment. Then, the medium was replaced with 100 μL fresh medium containing 10 μL Cell Counting Kit-8 (CCK8) reagent (Dojindo, CK04), and cells were incubated at 37 °C for 2 h. Cell viability was measured at 450 nm absorbance using a microplate reader (Thermo Scientific, Multiskan FC).

To evaluate the combination effect of dalpiciclib and chidamide at indicated concentrations, a series of doses and effects were entered into the CompuSyn software tool for treatment combination analysis. For each treatment alone and their combinations (constant ratio combinations), the software automatically calculated CI values at ED50 to estimate the combination effect. The resulting CI theorem offered quantitative definition for additive effects (CI = 1), synergism (CI < 1), and antagonism (CI > 1) of drug combinations.

### Cell cycle and cell apoptosis analysis

Cell cycle analysis was performed according to the instructions of the Cell Cycle Detection Kit (Biosharp, BL114A). Harvested cells were fixed in 70% ethanol for 2 h at 4 °C. After washing, the fixed cells were incubated in working solution containing 1 mg/mL RNase and 50 μg/mL PI at 37 °C for 30 min. The stained cells were centrifuged and re-suspended in PBS for flow cytometry (FCM) analysis. Cell apoptosis was evaluated using an Annexin V-FITC Apoptosis Assay Kit (Biosharp, BL110A). Briefly, the harvested cells were stained in 100 μL binding buffer containing 5 μL annexin V/FITC and 5 μL PI for 20 min at room temperature (RT). Then, cells were centrifuged and re-suspended in PBS, and FCM analysis was performed within 1 h.

### Western blotting

Cells were harvested and lysed using RIPA buffer containing 1% PMSF (Solarbio, P0100, 1:1000). Protein lysates were separated by SDS-PAGE gel electrophoresis, transferred onto NC membranes, and blocked with 10% skim milk in TBST. Membranes were incubated with primary antibodies against phosphor-retinoblastoma (S807) (pRb) (Abmart, T55499S, 1:500), retinoblastoma (Rb) (Abmart, T55661S, 1:1000), cyclin E2 (Selleck, F2373, 1:1000), cyclin D1 (Selleck, F0137, 1:1000), CDK4 (Selleck, F0322, 1:1000), CDK2 (Selleck, F0022, 1:1000), Bax (Selleck, F0037, 1:1000), p21 (Selleck, F0022, 1:1000), or β-actin (Abmart, P30002, 1:2000). After washing, membranes were incubated with HRP-conjugated secondary mouse & rabbit antibody (Abmart, M21003, 1:1000). Protein bands were visualized using the Bio-Rid ChemiDoc Touch Imaging System. Uncropped and unprocessed scans of the key Western blot images are provided in the Source Data file.

### Patient-derived organoids

BC organoids were surgically resected from Chinese PLA General Hospital, with the approval of the Ethics Institutional Review Committees of the Chinese PLA General Hospital and informed consent of the enrolled participants. Fresh BC tissues were cut into 0.2–0.5 mm^3^ pieces and digested in AdDF + ++ buffer (Advanced DMEM/F12 containing 1× Glutamax, 5 μM Y-27632, and antibiotics) containing 100 μg/ml type IV collagenase (Sigma-Aldrich, C5138) at 37 °C for 20 min. The digestion suspension was then strained over a 100 μm filter, centrifuged at 300 *g* for 5 min. and resuspended in cold matrix gel (Corning, 354234), seeded into prewarmed 24-well suspension culture plates (BIOFIL, TCP010024) for gelation, and added BC organoid medium (following the formula reported by Sachs, et al.^37^) 500 μL per well. The organoids were incubated at 37 °C/5% CO_2_ incubator, and medium was changed every 3 days and observed through a Nikon Ti2 microscope.

### Live/dead staining

Following BC organoid incubation with dalpiciclib, chidamide, or the combination of dalpiciclib and chidamide, live or dead cells were analyzed by Live-Dead Cell Staining Kit (ApexBio, K2081). Briefly, the organoid samples were transferred to centrifuge tubes with 1 mL pipette tips and incubated with a reagent working solution for 30 min. The live and dead cells were stained by green and red fluorescence, respectively. The confocal imaging stacks were collected with a Z-step mode using a Zeiss LSM 710 (Carl Zeiss). Three-dimensional reconstructions were processed offline using ZEN (Carl Zeiss).

### Immunohistochemistry (IHC)

Organoids were fixed in 4% paraformaldehyde followed by dehydration, paraffin embedding, and sectioning. IHC was performed using antibodies against ERa (ZSGB-BIO, TA506414) and HER2 (ZSGB-BIO, ZM-0065). Images were acquired on a Nikon Ti2 microscope.

### Statistics and reproducibility

This is a single-arm clinical trial utilized BOIN design to determine the MTD. Sample size calculation for this study was conducted at http://www.trialdesign.org. The target DLT rate (defined as the number of patients experiencing DLT at the current dose /the total number of evaluable patients treated at the same dose) is set at 0.33. The first treatment cycle was used as the DLT observation window to determine dose escalation or de-escalation. Each dose group could enroll up to 12 patients, with a maximum of 30 patients for the entire study. Descriptive statistics were used to analyze clinical characteristics, efficacy, and safety. Results were presented as counts (percentages), mean ± standard deviation, or median (minimum, maximum). Survival curves were generated using the Kaplan–Meier method, and 95% confidence intervals were calculated using the Brookmeyer–Crowley method. Statistical analysis was performed using SAS 9.4 software. No data were excluded from the analyses. The experiments were not randomized. The Investigators were not blinded to allocation during experiments and outcome assessment. Subgroup analyses and biomarker analyses were not prespecified in the study protocol and were conducted post hoc for descriptive purposes only.

### Reporting summary

Further information on research design is available in the Nature Portfolio Reporting Summary linked to this article.

## Supplementary information

Supplementary Information

Reporting Summary

Transparent Peer Review file

## Source data

Source Data 1

Source Data 2

## Data Availability

To protect participant confidentiality and comply with intellectual property agreements, individual deidentified data supporting the findings of this study will be made available upon request, beginning 24 months after the completion of the study. Qualified researchers should submit a detailed research proposal to the corresponding author at wangtao733073@163.com, outlining the purpose and intended use of the data. The data sharing request will be reviewed by the lead clinical institution and study sponsor to ensure alignment with applicable confidentiality and intellectual property policies. A response will be provided within two weeks. Data sharing will be contingent upon signing a data access agreement with the sponsor. The full study protocol and statistical analysis plan are provided in the Supplementary Information. Source data are provided with this paper.
