# Supplementary material for: Dalpiciclib plus chidamide in HR + /HER2−advanced breast cancer after CDK4/6 inhibitor failure: a phase Ib trial
Source: Nat Commun. 2026 Mar 20;17:5177. doi: 10.1038/s41467-026-70650-6 (PMC13254248; doi:10.1038/s41467-026-70650-6)
Supplement: Supplementary file 1 — Supplementary Information [file 41467_2026_70650_MOESM1_ESM.pdf]

Table S1. Dose-limiting toxicities.

Hematologic Toxicity	Neutropenia lasting ≥ 5 days
	Grade 4 thrombocytopenia, or grade 3 thrombocytopenia with clinically significant bleeding.
	Neutropenia ≥ grade 3 associated with fever (≥ 38.0°C lasting for 1 hour or > 38.3°C).
	Anemia ≥ grade 4.
Non-Hematologic Toxicity	Any non-hematologic toxicity ≥ grade 3, except for the following:<br><br>Grade 3-4 nausea/vomiting and/or diarrhea and/or electrolyte disturbances, which resolve to ≤ grade 2 within 72 hours after optimal supportive treatment.<br><br>Clearly tumor-related, drug-unrelated grade 3-4 elevations in alkaline phosphatase or gamma-glutamyl transferase (GGT).

Table S2. The principles of dose titration during the study.

DLT rate	Strategy
Estimated DLT rate was ≤ 26%	Increase the daily dose to the next higher level.
Estimated DLT rate > 39.5%	De-escalated to the next lower level.
> 26% and ≤ 39.5%	Maintain the current daily dose.

DLT, dose limit toxicity.

Table S3. Incidence of adverse events occurring in each group.

Adverse Events	A		B		C		D		ALL	
	Any Grade	Grade ≥ 3	Any Grade	Grade ≥ 3	Any Grade	Grade ≥ 3	Any Grade	Grade ≥ 3	Any Grade	Grade ≥ 3
Neutrophil count decreased	4(100%)	4(100%)	3(100%)	3(100%)	12(100%)	12(100%)	3(100%)	3(100%)	22(100%)	22(100%)
White blood cell counts decreased	4(100%)	1(25%)	3(100%)	3(100%)	12(100%)	9(75%)	3(100%)	1(33.3%)	22(100%)	14(63.6%)
Platelet count decreased	4(100%)	3(75%)	3(100%)	2(66.7%)	10(83.3%)	2(16.7%)	2(66.7%)	0	19(86.4%)	7(31.8%)
Anemia	4(100%)	2(50%)	3(100%)	0	9(75%)	1(8.3%)	3(100%)	1(33.3%)	19(86.4%)	4(18.2%)
Alanine aminotransferase increased	2(50%)	0	2(66.7%)	0	2(16.7%)	0	0	0	6(27.3%)	0
Aspartate aminotransferase increased	2(50%)	0	1(33.3%)	0	4(33.3%)	0	0	0	7(31.8%)	0
Blood alkaline phosphatase increased	0	0	1(33.3%)	0	3(25%)	0	0	0	4(18.2%)	0
Hypoalbuminemia	2(50%)	0	2(66.7%)	0	3(25%)	0	1(33.3%)	0	8(36.4%)	0
Hypokalemia	3(75%)	0	1(33.3%)	0	2(16.7%)	0	1(33.3%)	0	7(31.8%)	0
Fatigue	4(100%)	0	1(33.3%)	0	7(58.3%)	0	1(33.3%)	0	13(59.1%)	0
Nausea	2(50%)	0	0	0	4(33.3%)	0	0	0	6(27.3%)	0
Vomiting	2(50%)	0	0	0	1(8.3%)	0	0	0	3(13.6%)	0

Adverse Events	A		B		C		D		ALL	
	Any Grade	Grade ≥ 3	Any Grade	Grade ≥ 3	Any Grade	Grade ≥ 3	Any Grade	Grade ≥ 3	Any Grade	Grade ≥ 3
Decreased appetite	1(25%)	0	1(33.3%)	0	3(25%)	0	0	0	5(22.7%)	0
Hematochezia	1(25%)	0	0	0	1(8.3%)	1(8.3%)	0	0	2(9.1)	1(4.5%)

Fig S1

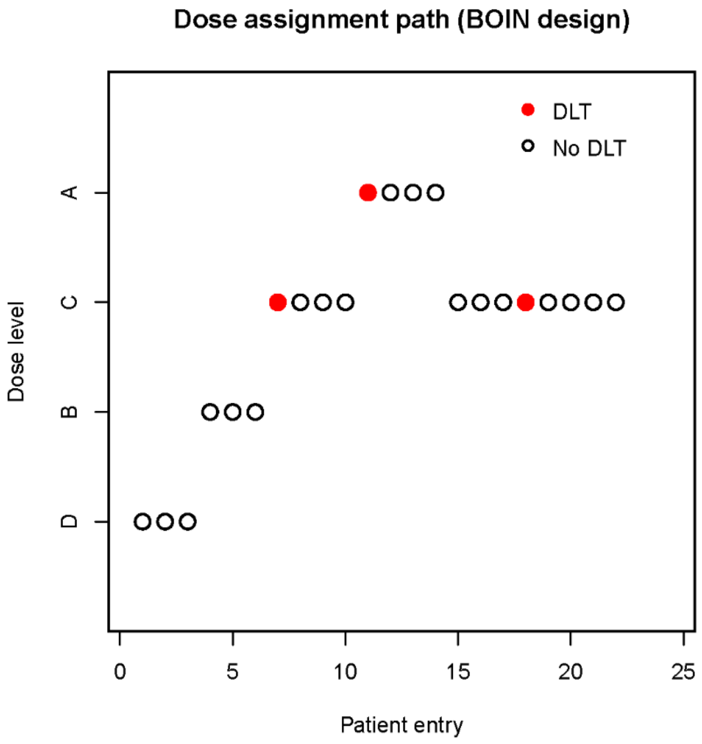

Supplemental Figure 1. Dose escalation path based on BOIN design. Each dot represents an individual patient. Source data are provided as a Source Data file.

Fig S2

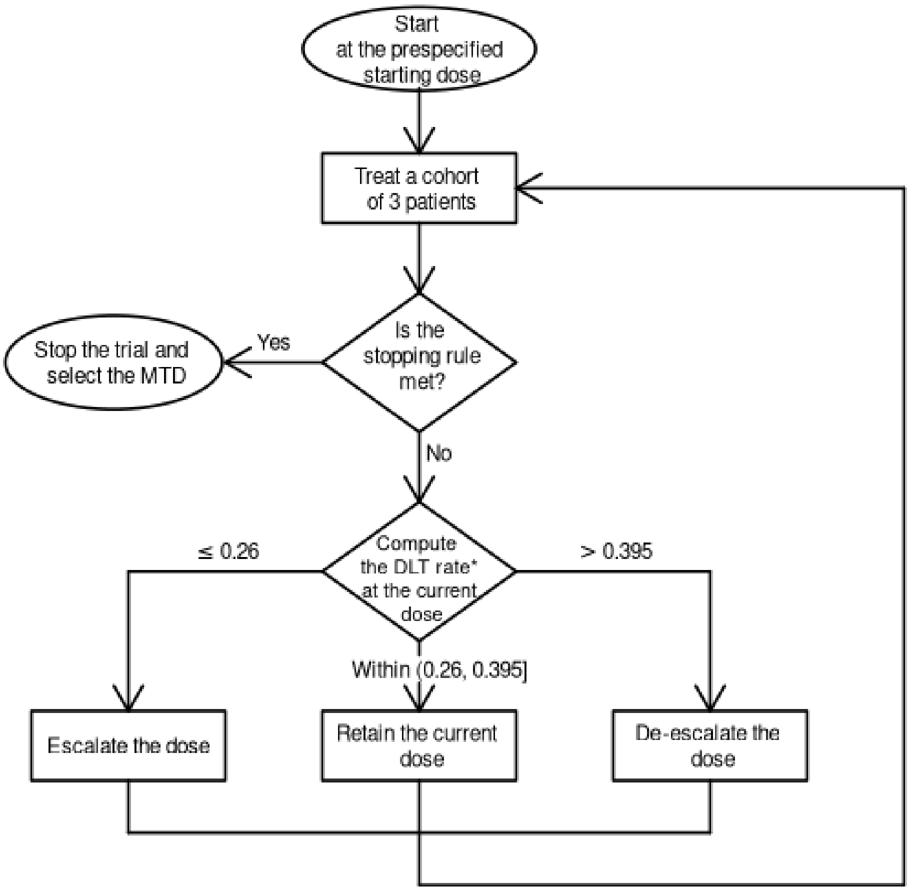

$$\text{* DLT rate} = \frac{\text{Total number of patients who experienced DLT at the current dose}}{\text{Total number of evaluable patients treated at the current dose}}$$

Supplemental Figure 2. The Bayesian optimal interval design.

Figure S3

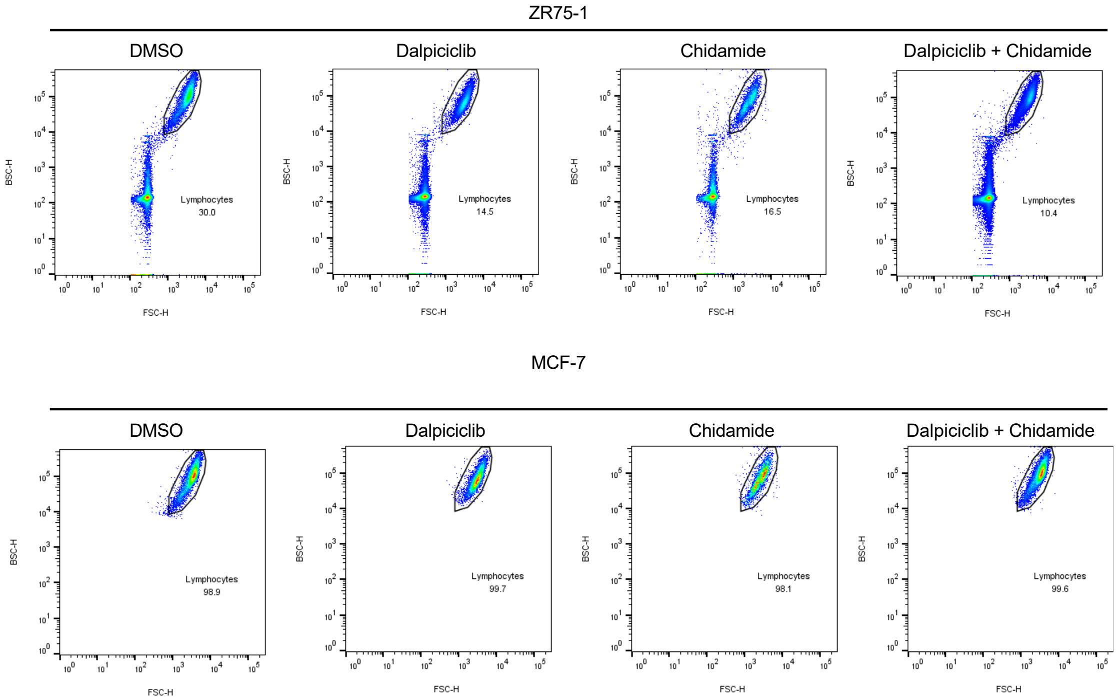

Supplemental Figure 3. The FACS sequential gating strategies.

PROTOCOL

Exploration of Dalpiciclib plus Chidamide in HR+/HER2- advanced breast cancer after failure of CDK4/6 inhibitor: a phase Ib study

Protocol version: v. 1.0

Protocol Date: August 28, 2022

Protocol No.: MA-BC-II-047

Site in Charge: The Fifth Medical Center of the Chinese People's Liberation Army General Hospital

Principal investigator: Prof. Tao Wang

Table of Contenets

Table of Contenets 4
Synopsis 6
Schedule of Activities 15
1. Study Background and Scientific Rationale 26
1.1. Study Background 26
1.2. Scientific Rational 27
2. Objective and Endpoints 30
2.1. Objective 30
2.2. Endpoints 30
2.2.1. Primary endpoint 30
2.2.2. Secondary endpoints 31
2.2.3. Exploratory endpoints 31
3. Study Design 31
4. Subject Selection and Withdrawal 32
4.1. Inclusion criteria 32
4.2. Exclusion criteria 33
4.3. Criteria for the termination of treatment 34
4.4. Criteria for the termination of study 34
5. Study Intervention 35
5.1. Study Intervention Administraion 35
5.2. Dose Modifiction 35
5.3. Concomitant medication and concomitant therapy 37
6. Study Procedures 38
6.1. Baseline period 39
6.2. Treatment period 40
7. Study Assessments 41
7.1. Safety Assessments 41
7.1.1. Pregnancy Testing 41
7.1.2. Adverse event 41
7.1.3. Laboratory safety evaluation 41
7.2. Effectiveness Assessments 41
8. Adverse Events 43
8.1. Defination of Adverse Events 43
8.1.1. Adverse Events 43
8.1.2. Serious Adverse Events 44
8.2. Classification of Adverse Events 44
8.2.1. Ralationship of Adverse Events 44
8.2.2. Severity of Adverse Events 45
8.3. Collection and Follow-up of Adverse Events 45
8.4. Reporting Serious Adverse Events 46
8.5. Pregnancy 46
8.6. Disease Progression and Death 47
9. Statistical Analysis Methods 48

9.1. Sample Size Determination....48
9.2. Analysis Population....48
9.3. Statistical Analyses....49
9.3.1. General Methods....49
9.3.2. Subject Disposition....49
9.3.3. Demographics and baseline characteristics....49
9.3.4. Safety Analysis....49
9.3.5. Efficacy Analyses (Secondary)....50
10. Research management....50
10.1. Compliance with Ethical and Regulatory Guidelines....50
10.2. Institutional and Ethics Norms....50
10.3. Informed Consent....51
10.4. Privacy....51
10.5. Quality Assurance and Control....51
10.6. Data Management....52
10.6.1. Data Collection and Quality Control....52
10.6.2. Data Storage....52
10.7. Protocal Revision....53
11. Reference....54

Synopsis

**Study Title**	**Exploration of Dalpiciclib plus Chidamide in HR+/HER2- advanced breast cancer after failure of CDK4/6 inhibitor: a phase Ib study**
**Protocol Number**	MA-BC-II-047
**Version/Revision**	1.0
**Document Date**	August 28, 2022
**Sponsor-Investigator Site**	**The Fifth Medical Center of the Chinese People's Liberation Army General Hospital**
**Principal investigator**	**Tao Wang**
**Study objects**	**Patients with HR+/HER2- advanced breast cancer**
**Objectives**	**Exploration of maximum tolerated dose (MTD) of Dalpiciclib plus Chidamide**

<table>
<tr><td>Study Endpoints</td><td>Primary endpoint:
    Exploration of MTD of Dalpiciclib plus Chidamide
Secondary endpoints:
    Objective response rate of different dose groups
    Safety of different dose groups (incidence of treatment-related adverse events)
    PFS of different dose groups</td></tr>
<tr><td>Sample Size</td><td>The Bayesian Optimal Interval (BOIN) design will be employed for dose escalation. Each dose cohort will enroll a maximum of 12 subjects, with an overall maximum sample size of 30 subjects for the entire study.</td></tr>
<tr><td>Study Design</td><td>A phase 1B study to explore the MTD of dalpiciclib + chidamide in HR+/HER2- advanced breast cancer after the failure of CDK4/6 inhibitor therapy.
Dalpiciclib will be administered in a dose of 100 mg/d or 125 mg/d. Chidamide shall be designed in a dose of 25 mg/BIW or 20 mg/BIW.
<table>
<tr><td>Dose group</td><td>Dalpiciclib</td><td>Chidamide</td></tr>
<tr><td>Group A</td><td>125 mg/d</td><td>25 mg/BIW</td></tr>
<tr><td>Group B</td><td>125 mg/d</td><td>20 mg/BIW</td></tr>
<tr><td>Group C</td><td>100 mg/d</td><td>25 mg/BIW</td></tr>
<tr><td>Group D</td><td>100 mg/d</td><td>20 mg/BIW</td></tr>
</table>
(Table 1)
Bayesian optimal interval (BOIN) design method will be used in this clinical trial (Yuan et al,2016; Jin Liu et al., 2018) to determine the maximum tolerated dose (MTD). Given the target</td></tr>
</table>

<table>
<tr>
<td></td>
<td>DLT rate of 33% for the combination treatment, the BOIN design uses the following optimization rules to make dose fluctuation decisions: if the estimated DLT rate is ≤ 0.26 at the current dose, then escalate to the next higher dose level; if the estimated DLT rate is >0.395 at the current dose, de-escalate to the next lower dose level; otherwise, stay at the current dose level. If the number of patients assigned to single dose reaches twelve and the decision is to stay, then the trial will be stopped. Thus the maximum patient number of each dose level is twelve, or otherwise the trial will also be terminated; and the maximum total planned sample size is thirty. And whether to stay, escalate or de-esclate will also depends on the available efficacy evidence besides safety.

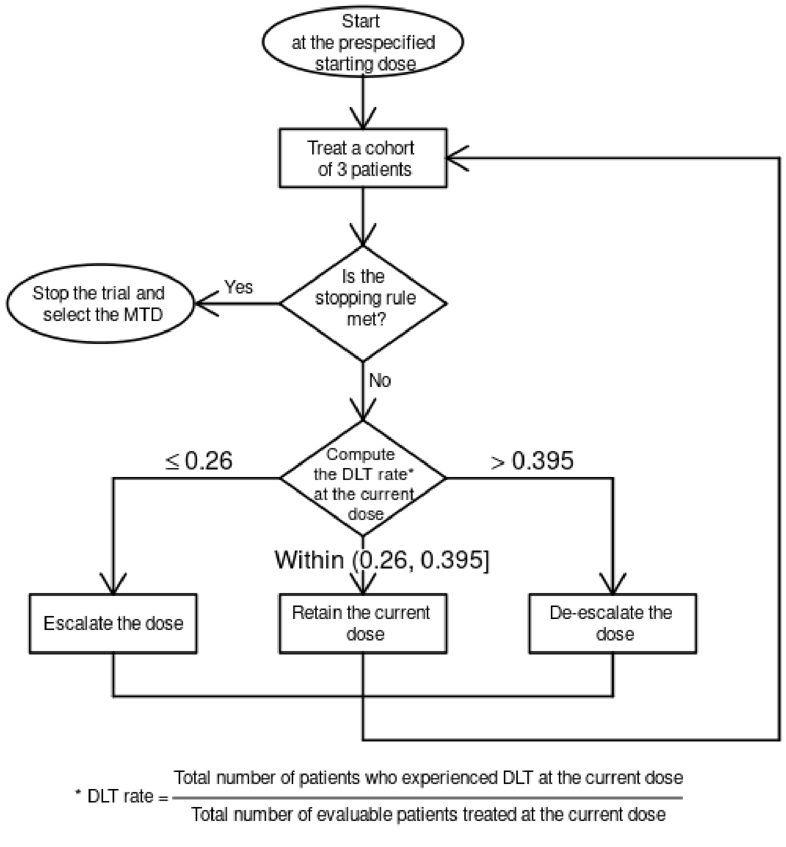

(Figure 1)</td>
</tr>
<tr>
<td>**Treatment**</td>
<td>• **Dalpiciclib: po., qd, administered on an empty stomach (fasting required ≥1 hour before and after dosing). The drug will be given orally once daily for 3 weeks, followed by**</td>
</tr>
</table>

	**1 week off in each 4-week cycle.**<br>• **Chidamide: po., q2w. The interval between doses should not be less than 3 days (e.g. Monday and Thursday, Tuesday and Friday, Wednesday and Saturday, etc.), administered 30 minutes after meals.**<br>• **Endocrine therapy medication (doctor's choice)**<br>(1) Letrozole 2.5 mg po., qd;<br>(2) Anastrozole 1 mg po., qd;<br>(3) Exemestane 25 mg po., qd;<br>(4) Fulvestrant 500 mg administered intramuscularly on Cycle 1, Days 1 and 15, and then on Day 1 of every 28-day cycle
**Dose-limiting Toxicity (DLT)**	**DLT will be defined as any of the following drug-related or potentially drug-related adverse events occurring during the first cycle (per CTC-AE V5.0):**<br>**1. Hematological toxicity:**<br>• **Grade 4 neutropenia lasted for ≥5 days;**<br>• **Grade 4 thrombocytopenia, or grade 3 thrombocytopenia with clinically significant bleeding;**<br>• **Grade ≥ 3 neutropenia with fever (≥ 38.0 °C for 1 hour or > 38.3 °C);**<br>• **Grade ≥ 4 anemia.**<br>**2. Non-hematological toxicity:**<br>**Any grade ≥ 3 non-hematological toxicity, except:**<br>• **Grade 3-4 nausea/vomiting and/or diarrhea and/or electrolyte disturbances that have recovered to grade ≤ 2 within 72 hours after optimal supportive treatment;**<br>• **There will be a definite increase of grade 3-4 alkaline phosphatase and glutamyl transpeptidase related to tumor but not related to treatment.**
**Inclusion Criteria**	**1. Patients have signed informed consent.**<br>**2. Female, aged ≥ 18 years.**

	**3. ECOG performance status: 0-2.**<br><br>**4. Life expectancy ≥ 6 months.**<br><br>**5. Histological or cytological confirmation of estrogen receptor positive and/or progesterone receptor positive(≥ 10%), HER2-negative locally advanced or metastatic breast cancer not amenable to curative resection or radiotherapy.**<br><br>**6. Prior antitumor therapy: 1) previously received ≤1 line of chemotherapy for recurrent or metastatic breast cancer; 2) Disease recurrence and/or metastasis during or after treatment with Palbociclib or Abemaciclib or Ribociclib in the setting of (neo-)adjuvant therapy, or disease progression during treatment with palbociclib or Abemaciclib or Ribociclib in the metastatic setting ; 3) ≤3 prior lines of endocrine therapy for metastatic disease;**<br><br>**7. At least one extracranial measurable lesion as defined by RECIST v1.1;**<br><br>**8. Adequate organ and bone marrow function;**<br><br>• Neutrophil count ≥ 1.5 × 10^9/L;<br>• Platelets ≥ 90 × 10^9/L;<br>• Hemoglobin ≥ 90g/L;<br>• Total bilirubin (TBIL) ≤ 1.5 × ULN;<br>• ALT and AST ≤ 2.5 × ULN;<br>• Urea/blood urea nitrogen (BUN) and creatinine (Cr) ≤1.5×ULN;<br>• Left ventricular ejection fraction (LVEF) ≥ 50%;<br>• The QT interval corrected by the Fridericia formula (QTcF) is < 470 ms.<br>**INR ≤ 1.5 × ULN, APTT ≤ 1.5 × ULN.**

	**9. Patients recovers from AE related to previous antitumor therapy before the first administration of the study drug (Grade ≤ 1)**
**Exclusion Criteria**	1. Previously received treatment with histone deacetylase inhibitor (HDACi);<br>2. Previously received Dalpiciclib;<br>3. Leptomeningeal metastasis confirmed by MRI or lumbar puncture ;<br>4. Radiologically confirmed CNS metastases; The following conditions will be excluded: 1) asymptomatic brain metastases not requiring immediate radiotherapy or surgery; 2) Previously received local treatment (radiotherapy or surgery) for brain metastases, stable for at least 4 weeks, and no symptomatic treatment (including glucocorticoids, mannitol, bevacizumab, etc.) for more than 2 weeks;<br>5. Patients with visceral crisis (such as lymphangitis carcinomatosis, bone marrow metastasis, leptomeningeal metastasis, diffuse liver metastasis with abnormal liver function), rapid disease progression, and that is not suitable for endocrine therapy;<br>6. Patients had ascites, pleural effusion and pericardial effusion with clinical symptoms at baseline, which required drainage within 4 weeks before the first medication;<br>7. Inability to swallow, intestinal obstruction, or other factors that affect medication administration and absorption;<br>8. Patients who are diagnosed with any other malignancy within 5 years prior to the study, excluding non-melanoma skin cancer treated with radical therapy, basal or squamous cell skin cancer or carcinoma in situ of the cervix and papillary thyroid.<br>9. Patients has undergone major surgery or major trauma or is expected to undergo major surgery within 4 weeks prior to treatment initiation;<br>10. Allergy to the any active component or excipient of the

	investigational drug(s) in this protocol;
**Criteria for the termination of treatment**	Patients should withdraw/discontinue treatment if any of the following situation occurs during the study:<br>1. Patients withdrew informed consent and requested withdrawal;<br>2. Disease progression;<br>3. Patients who cannot tolerate toxicity;<br>4. Patients who seriously violated the Protocol and should be terminated as assessed by the investigator;<br>5. Lost to follow-up or experienced pregnancy events;<br>6. Other investigator-determined reasons for withdrawal.
**Criteria for the termination of study**	Study termination criteria include, but are not limited to:<br>1. Identification of unanticipated, clinically significant, or unacceptable risks to participants;<br>2. The study drug/trial treatment is ineffective, or continuing the trial is futile;<br>3. The investigator decided to terminate the study for reasons such as significant delay in recruitment or major protocol violations.
**Safety Assessment**	Severity of adverse events are graded by CTCAE v5.0. Complete AE documentation must include: onset date, CTCAE grade, duration, intervention, and final outcome.
**Effectiveness Assessment**	**Participants will undergo weekly (±2-day window) monitoring of blood counts and liver/kidney function during the treatment period Tumor assessments by imaging must be conducted every 2 cycles (±7 days) until PD or new antitumor treatment, using RECIST 1.1 for response evaluation.After disease progression or initiation of new antitumor therapy, survival follow-up will be conducted every 12 weeks**
**Statistical Methods**	**The results of this trial are mainly analyzed by descriptive statistics. For measurement data, descriptive statistics**

	**including the mean, standard deviation, median, maximum, and minimum values were reported.**<br>**For categorical and ordinal data, frequencies (with percentages), rates, and corresponding confidence intervals were presented. All statistical analyses will be conducted using SAS 9.2 or above.**<br>**Safety analysis:**<br>**Descriptive statistical analysis shall be used to analyze the adverse events in each dose cohort.**<br>**Laboratory evaluation:**<br>**Laboratory parameters will be summarized by treatment group and study visit. The abnormality for each laboratory parameter will be assessed by investigator. A shift table from baseline to the worst post-baseline assessment according to the investigator's evaluation for each parameter listed below will be presented.**<br>**Other safety data:**<br>**Descriptive statistics of physical examination, electrocardiogram, and vital signs will be summarized according to the time point specified in the protocol and the dose group.**<br>**Efficacy analyses:**<br>**Progression-free survival (PFS) will be estimated using the Kaplan-Meier method, with survival curves plotted and the median time and 95% confidence interval provided. The objective response rate (ORR) and disease control rate (DCR) will be calculated along with the incidence rate and its 95% confidence interval.**
**End of the**	**24 months after enrollment of the final participant or the time**

**Study**	**when the investigator deemed it's necessary to end the trial earlier.**
**Study Duration**	**It is planned to be conducted from November 2022 to November 2024.**

Schedule of Activities

Items	Screening period		Treatment period (28 days for a treatment cycle)						Post-treatment		Survival follow-up
			The first cycle				≥ 2 cycles				
	D-28 to D-1	D-7 to D-1	D7	D14	D21	D28	D14	D28	Termination of treatmetn/withdrawl	Visit after the termination of treatment (4 weeks after the last administration)	q12w
			±2d	±2d	±2d	±2d	±2d	±2d			(±7d)
**Baseline Data**											
Sign Informed consent	×										
Demographics	×										

Items	Screening period		Treatment period (28 days for a treatment cycle)						Post-treatment		Survival follow-up
	D-28 to D-1	D-7 to D-1	The first cycle				≥ 2 cycles				
			D7	D14	D21	D28	D14	D28	Termination of treatmetn/withdrawl	Visit after the termination of treatment (4 weeks after the last administration)	q12w
			±2d	±2d	±2d	±2d	±2d	±2d			(±7d)
History of tumor/other diseases [1]	×										
Concomitant medications [2]	×		×								
**Laboratory Tests**											

Items	Screening period		Treatment period (28 days for a treatment cycle)						Post-treatment		Survival follow-up
	D-28 to D-1	D-7 to D-1	The first cycle				≥ 2 cycles				
			D7 ±2d	D14 ±2d	D21 ±2d	D28 ±2d	D14 ±2d	D28 ±2d	Termination of treatmetn/withdrawl	Visit after the termination of treatment (4 weeks after the last administration)	q12w (±7d)
Blood Routine [3]		×	×	×	×	×	×	×	If not done within 7 days	If indicated	
Urine Routine [4]		×	Once every 3 cycles						If not done within 7 days	If indicated	
Stool Routine [5]		×	Once every 3 cycles						If not done within 7 days	If indicated	

Items	Screening period		Treatment period (28 days for a treatment cycle)						Post-treatment		Survival follow-up
			The first cycle				≥ 2 cycles				
	D-28 to D-1	D-7 to D-1	D7 ±2d	D14 ±2d	D21 ±2d	D28 ±2d	D14 ±2d	D28 ±2d	Termination of treatmetn/withdrawl	Visit after the termination of treatment (4 weeks after the last administration)	q12w (±7d)
**Blood chemistry** [6]		×	×		×			×	If not done within 7 days	If indicated	
**Hepatitis B, Hepatitis C and HIV tests** [7]	×										
**Pregnancy tests** [8]		×								If indicated	
**Clinical evaluation and examination**											

Items	Screening period		Treatment period (28 days for a treatment cycle)						Post-treatment		Survival follow-up
			The first cycle				≥ 2 cycles				
	D-28 to D-1	D-7 to D-1	D7 ±2d	D14 ±2d	D21 ±2d	D28 ±2d	D14 ±2d	D28 ±2d	Termination of treatmetn/withdrawl	Visit after the termination of treatment (4 weeks after the last administration)	q12w (±7d)
Adverse events [9]	From the signing of informed consent to 28 days after the last dose										
Vital signs [10]		×	×		×			×	If not done within 7 days	×	
Physical examination [11]		×	×		×			×	If not done within 7 days	×	
ECOG PS score		×	×		×			×	If not done within 7 days	×	

Items	Screening period		Treatment period (28 days for a treatment cycle)						Post-treatment		Survival follow-up
			The first cycle				≥ 2 cycles				
	D-28 to D-1	D-7 to D-1	D7	D14	D21	D28	D14	D28	Termination of treatmetn/withdrawl	Visit after the termination of treatment (4 weeks after the last administration)	q12w
			±2d	±2d	±2d	±2d	±2d	±2d			(±7d)
ECG [12]		×	×		×			×	If not done within 7 days	If indicated	
Echocardiography [13]	×		Once every 3 cycles						If not done within 4 weeks	If indicated	
**Study drug**											
Endocrine therapy [14]			Take according to the label chosen by your doctor.								

Items	Screening period		Treatment period (28 days for a treatment cycle)						Post-treatment		Survival follow-up
	D-28 to D-1	D-7 to D-1	The first cycle				≥ 2 cycles				
			D7	D14	D21	D28	D14	D28	Termination of treatmetn/withdrawl	Visit after the termination of treatment (4 weeks after the last administration)	q12w
			±2d	±2d	±2d	±2d	±2d	±2d			(±7d)
Chidamide [15]			Take Chidamide within 30 minutes after meals, po., twice a week.								
Dalpiciclib [16]			Take on an empty stomach for 3 weeks and stop for 1 week, po., Qd.								
**Efficacy assessment**											
Imaging Assessment[17]	×		Imaging assessments will be performed every 2 cycles (±7 days) until disease progression or the initiation of a new anti-tumor therapy.								

Items	Screening period		Treatment period (28 days for a treatment cycle)						Post-treatment		Survival follow-up
	D-28 to D-1	D-7 to D-1	The first cycle				≥ 2 cycles				
			D7	D14	D21	D28	D14	D28	Termination of treatmetn/withdrawl	Visit after the termination of treatment (4 weeks after the last administration)	q12w
			±2d	±2d	±2d	±2d	±2d	±2d			(±7d)
**Follow-up after the termination of the treatment**											
Time to progression [18]									Until disease progression or initiation of new antitumor therapy		
Survival follow-up [19]											×

Note:

[1] History of tumor/other diseases: pathological results, ER/PR/HER2 status; History of cancer surgery, chemotherapy, radiotherapy

and treatment of other diseases; History of tumors other than breast cancer.

[2] Concomitant medication and concomitant therapy will be recorded within 28 days before the start of medication and during the study. Once a participant discontinues trial treatment, only concomitant medication and concomitant therapy should be recorded for new or unresolved adverse events related to trial treatment.

[3] Blood routine: hemoglobin, red blood cell, white blood cell, neutrophil count, lymphocyte count and platelet count.

[4] Urine routine: urine protein, urine sugar, urine occult blood (urine red blood cells, white blood cells). 24-hour urine protein quantification shall be performed if the semi-quantitative method showed protein 2+.

[5] Stool routine: including fecal occult blood.

[6] Blood biochemistry: total bilirubin, conjugated bilirubin, ALT, AST, AKP, γ-GT, LDH, total protein, albumin, urea/blood urea nitrogen, creatinine, uric acid, fasting blood glucose, triglyceride, cholesterol, potassium, sodium, chlorine, calcium, phosphorus, magnesium; Additional myocardial zymogram examination if indicated.

[7] Hepatitis B, Hepatitis C and HIV tests: five tests for hepatitis B. Viral replication (HBV DNA) test should be performed if the results are abnormal; Hepatitis C virus antibody (anti-HCV), HIV antibody test.

[8] Pregnancy test: Serum pregnancy test should be performed within 1 week before the first medication for women of childbearing potential.

[9] Adverse events: Adverse events will be recorded from the signing of informed consent until at least 28 days after the last medication, and shall be followed up until the remission or stabilization of adverse events, or until the initiation of new tumor therapy.

[10] Vital signs: temperature, respiration, pulse, blood pressure.

[11] Physical examination and weight measurement: the main body of the system to check (facial lymph nodes, skin, head, eye, ear, mouth, respiratory system, cardiovascular system, abdomen, reproductive and urinary system, musculoskeletal, neurological and mental state), screening and research at the end of the document comprehensive physical examination results, only record abnormal situation during test.

[12] 12-lead ECG: If an abnormal ECG is found to be clinically significant, the investigator may reconfirm it if deemed necessary.

[13] Echocardiography: Follow up LVEF value changes, such as LVEF decreased to < 50% and ≥10% from baseline, or chest pain, palpitation and other symptoms, unplanned examination can be added.

[14] Endocrine therapy: letrozole, oral, 2.5 mg, once a day, given under fasting, continuous administration for 28 days as a cycle; Fulvestrant 500mg, D1 (with an additional dose on D15 of Cycle 1 only), intramuscular injection, 28 days a cycle.

[15] Chidamide: po., twice a week. The interval between doses should not be less than 3 days (e.g. Monday and Thursday, Tuesday and Friday, Wednesday and Saturday, etc.), administered 30 minutes after meals.

[16] Dalpiciclib will be taken orally on an empty stomach, once a day, take for 3 weeks (D1-21), and hold for 1 week (D22-28), in a 28-day cycle. For Dalpiciclib to be administered on an empty stomach, orally take with warm water in the morning, and fast 1 hour before and 1 hour after the continuous administration of Dalpiciclib.

[17] Imaging: Screening imaging includes enhanced CT or MRI of the chest and abdomen, as well as CT/MRI of other sites (such as the neck, pelvis, or brain) where investigators suspect the presence of lesions. Baseline tumor assessment can be extended to 4 weeks prior to first dose administration, and CT/MRI scan results obtained before signing informed consent can be used for screening

tumor assessment as long as they meet the requirements; Bone scanning should be performed when bone metastases are suspected clinically. Imaging examination during the treatment period should be performed under the same conditions as the baseline examination (thickness of the scan, use of contrast media, etc.), 6 cycles before medication, every 2 cycles for the lesions found at baseline (bone scan for suspected bone progression or CR confirmation), and then every 3 cycles; If new lesions are suspected, timely examination can be performed. The first PR/CR should be confirmed after 4-6 weeks. The allowable window period for the imaging schedule will be ±7 days. Unplanned imaging may be performed when disease progression (e.g., worsening symptoms) is suspected.

[18] Participants who terminated the trial treatment for reasons other than radiographically proven disease progression and did not undergo radiographic evaluation within 4 weeks before the end of the trial will be required to undergo radiographic evaluation at the end of the treatment and to continue tumor efficacy follow up at the prescribed frequency after the end of the trial until disease progression is documented or new tumor therapy is initiated.

[19] Survival follow-up: After the termination of trial treatment, survival status and subsequent antitumor treatment shall be collected every 3 months through clinical follow-up or telephone follow-up until death.

.

Study Background and Scientific Rationale

Study Background

With the advent of targeted therapies, the treatment landscape for patients with hormone receptor-positive (HR+), HER2-negative (HER2-) metastatic breast cancer (MBC) has significantly evolved. Cyclin-dependent kinase 4 and 6 (CDK4/6) inhibitors, in combination with endocrine therapy, have become the standard of care for both first-line treatment and following progression on prior endocrine therapy[1]. Numerous large randomized clinical trials (RCTs) have demonstrated that CDK4/6 inhibitors combined with endocrine therapy significantly improve progression-free survival (PFS) in patients with HR+/HER2- MBC [2-6]. Updated analyses have further demonstrated that endocrine therapy combined with dalpiciclib or ribociclib significantly improves overall survival (OS) in this patient population.

Currently, four CDK4/6 inhibitors—palbociclib, abemaciclib, ribociclib, and dalpiciclib —have been approved by regulatory agencies for the treatment of HR+/HER2- breast cancer. These inhibitors have all been approved in combination with endocrine therapy for advanced HR+/HER2- breast cancer, while abemaciclib has also been approved for use in the adjuvant setting for patients with early-stage HR+/HER2- breast cancer at high risk of recurrence, as well as for advanced disease.

At present, CDK4/6 inhibitors are the standard treatment for HR+ metastatic breast cancer . However, there is no established standard treatment following failure of CDK4/6 inhibitor . While these drugs have widespread clinical experience, our understanding of the effects of continued CDK4/6 blockade in patients who have previously received CDK4/6 inhibitors remains limited. Emerging data suggest that clinical resistance may be mediated by various mechanisms, including RB1 inactivation, overexpression of CDK6, CCNE1/2, and Aurora kinase A, as well as activating mutations in FGFR, ERBB2, AKT1, and the RAS oncogene family [7-11]. However, the clinical utility of these proposed biomarkers for routine medical decision-making

remains to be fully established. Despite the lack of definitive data, some clinicians have administered a second course of CDK4/6 inhibition following disease progression in the first-line or second-line treatment setting for patients with HR+/HER2− metastatic breast cancer.. This approach mirrors the longstanding practice of continuing HER2-targeted therapy after progression in HER2-positive breast cancer.

Scientific Rational

Dalpiciclib is a CDK4/6 kinase inhibitor developed by Jiangsu Hengrui Medicine Co., Ltd., classified as a novel Class 1.1 drug. Preclinical data show that dalpiciclib selectively inhibits CDK4/6 kinase activity, preventing its complex with Cyclin D from phosphorylating downstream Rb protein, thereby blocking the transition of cells from the G1 phase to the S phase. This mechanism inhibits cell proliferation and exerts antitumor effects. Dalpiciclib, either as a monotherapy or in combination with endocrine agents, exhibits significant antiproliferative effects on Rb-positive tumor cells in vitro (IC50: 60–350 nM). Moreover, SHR6390 has shown potent antitumor activity in multiple xenograft models in nude mice. Clinical studies also demonstrate that dalpiciclib, in combination with endocrine therapy, exerts strong antitumor effects in patients with advanced HR+/HER2− metastatic breast cancer (MBC).

A multicenter, randomized, controlled, double-blind phase III study—DAWNA-1 (study number: SHR6390-III-301)—led by Professor Xu Binghe, evaluated the efficacy and safety of dalpiciclib in combination with fulvestrant versus placebo plus fulvestrant in patients with advanced HR+/HER2- breast cancer who had previously relapsed or progressed on endocrine therapy[12]. A total of 361 patients were enrolled in the study, patients with recurrent or advanced disease who have received ≤1 prior line of chemotherapy are allowed. The sample was randomized in a 2:1 ratio, with the experimental group receiving 150 mg of dalpiciclib in combination with 500 mg of fulvestrant, and the control group receiving 150 mg of placebo in combination with 500 mg of fulvestrant. Stratification was based on the presence of visceral metastasis (yes or no) and menopausal status (postmenopausal or premenopausal). The study population for DAWNA-1 was 100% Chinese, with 27% of patients having received chemotherapy in the advanced stage, and 44% of patients being

premenopausal/perimenopausal. Data cutoff was March 25, 2022, and the results showed that dalpiciclib significantly improved patients′ survival. Compared with the placebo group, the median progression-free survival (PFS) was significantly longer in the dalpiciclib plus fulvestrant group, with 16.6 months (95% CI 15.2–18.6) versus 7.2 months (95% CI 5.6–9.2), respectively (HR = 0.50). A consistent trend of PFS benefit from dalpiciclib plus fulvestrant treatment was observed across all predefined subgroups (HR < 1). The objective response rate (ORR) in the dalpiciclib group was 35.7% (95% CI 29.6–42.7), compared to 23.3% (95% CI 16.1–31.9) in the placebo group. OS data were not yet mature, with 82 deaths (22.7%) reported. The evaluation results for the time to first chemotherapy showed that, compared to the placebo group, the median PFS in the dalpiciclib group was 24.0 months (95% CI 20.2-NR) for both first and subsequent chemotherapy, whereas the placebo group had a median PFS of 11.8 months (95% CI 9.0–14.5) to first chemotherapy. Treatment with dalpiciclib plus fulvestrant prolonged the time to subsequent first chemotherapy. Furthermore, the DAWNA-1 study demonstrated that dalpiciclib had a manageable and reversible safety profile. In the dalpiciclib group, grade 3 or 4 adverse events were primarily hematological, with the most common being neutropenia (86.3%) and leukopenia (69.6%), with no cases of febrile neutropenia reported. The median time to first onset of any grade neutropenia in the dalpiciclib group was 15 days. With increasing treatment cycles, the incidence and severity of neutropenia decreased. Additionally, the average duration of grade 3 or higher adverse events in the dalpiciclib group was only 3 days, and there were no reports of treatment interruption due to hematological adverse events.

The DAWNA-2 study (study number: SHR6390-III-302) explored the efficacy and safety of dalpiciclib in combination with letrozole/anastrozole as first-line treatment for HR+/HER2− locally advanced or metastatic breast cancer[13]. The experimental group received dalpiciclib in combination with letrozole/anastrozole, while the control group received placebo in combination with letrozole/anastrozole. Data cutoff was June 1, 2022, with a median follow-up of 21 months. The DAWNA-2 study met its primary endpoint in an interim analysis. The median progression-free survival (PFS) in the dalpiciclib group was significantly longer than in the placebo

group (30.6 months [95% CI: 30.6–NR] vs 18.2 months [95% CI: 16.5–22.5], HR = 0.51). The PFS assessed by the Independent Review Committee (IRC) was consistent with the investigator-assessed PFS. Both the investigator-assessed objective response rate (ORR) and the IRC-assessed ORR showed significant clinical benefit with the addition of dalpiciclib to letrozole/anastrozole. The investigator-assessed ORR was 57.4% (95% CI: 51.6–63.1) in the dalpiciclib group compared to 47.7% (95% CI: 39.6–55.9) in the placebo group (P = 0.0233). The IRC-assessed ORR was 62.4% (95% CI: 56.7–67.9) in the dalpiciclib group compared to 53.6% (95% CI: 45.4–61.7) in the placebo group (P = 0.0350). In terms of safety, the incidence of grade ≥3 adverse events (AEs) in the dalpiciclib group was 90.4%, with neutropenia (85.7%) and leukopenia (66.6%) being the most common AEs.

Therefore, a deeper understanding of the potential utility of ongoing CDK4/6 inhibition, particularly with dalpiciclib, following initial progression is crucial to address the unmet clinical needs in the current HR+ metastatic breast cancer (MBC) treatment landscape.

Chidamide targets HDAC1, HDAC2, and HDAC3 from class I, as well as HDAC10 from class IIb. HDAC1/2 are key regulators of p53, and the deacetylation of HDAC3 is associated with cell proliferation, DNA damage, and apoptosis. HDAC10 inhibits DNA damage. HDAC inhibitors (HDACi) modify histones such as p21, regulating the expression of cyclin D1, Cdk4, and Cdk6. Additionally, HDACi induce apoptosis by acetylating non-histone proteins such as p53, STAT3, and tubulin. The deacetylation of p53 by HDAC inhibitors further enhances its interaction with p21, leading to G0/G1 cell cycle arrest[14-16].

The approval of chidamide for use in breast cancer primarily stems from a multicenter, randomized, double-blind phase III clinical trial[5]. The study enrolled 365 postmenopausal women aged 18 to 75 years with hormone receptor-positive, HER2-negative, and advanced breast cancer who had relapsed or metastasized after prior endocrine (adjuvant or salvage) therapy. Patients were randomly assigned to receive chidamide in combination with exemestane or placebo in combination with exemestane. Treatment continued until disease progression or intolerable toxicity occurred. The primary endpoint was progression-free survival (PFS), while secondary endpoints included overall survival (OS), objective response rate (ORR), duration of response

(DOR), and clinical benefit rate (CBR). The results demonstrated that chidamide plus exemestane significantly improved PFS (9.2 months vs. 3.8 months), with superior ORR and CBR compared to the control group[17].

From a mechanistic perspective, the combination of HDAC inhibitors (HDACi) and CDK4/6 inhibitors may have a synergistic effect. Based on this evidence and hypothesis, and considering the lack of published studies on the combination of CDK4/6 inhibitors and HDAC inhibitors, as well as the absence of recommended doses, safety, and efficacy data for the dalpiciclib and chidamide combination regimen, this study aims to conduct a Phase Ib trial to explore the maximum tolerated dose (MTD) of the dalpiciclib in combination with chidamide.

Objective and Endpoints

Objective

- **To evaluate the safety and tolerability of the combination of an HDAC inhibitor (Chidamid), a CDK4/6 inhibitor (dalpiciclib), and endocrine therapy in patients with HR+/HER2− advanced breast cancer who have progressed after prior CDK4/6 inhibitor treatment. Based on toxicity and efficacy outcomes observed in trial participants, four dose levels will be assessed, and the optimal biological dose will be determined.**
- **To assess the efficacy and safety of dalpiciclib in combination with Chidamid and endocrine therapy in the treatment of HR+/HER2− advanced breast cancer following progression on prior CDK4/6 inhibitor therapy..**

Endpoints

Primary endpoint

- MTD of Dalpiciclib plus Chidamide: The highest dose level at which no more than one-third of subjects experience dose-limiting toxicity (DLT) during the

completion of one treatment cycle.

Secondary endpoints

- Objective response rate of different dose groups
- Disease control rate of different dose groups
- The incidence of adverse events (AEs) of different dose groups
- PFS of different dose groups.

Exploratory endpoints

- Potential predictive biomarker parameters related to efficacy in peripheral blood and tumor tissue specimens (e. g., PIK3CA, ESR1, etc.).

Study Design

This study is a single-arm, open-label, dose-escalation Phase Ib clinical trial.

Dalpiciclib will be administered in a dose of 100 mg/d or 125 mg/d. Chidamide shall be designed in a dose of 25 mg/BIW or 20 mg/BIW.

Dose group	Dalpiciclib	Chidamide
Group A	125 mg/d	25 mg/BIW
Group B	125 mg/d	20 mg/BIW
Group C	100 mg/d	25 mg/BIW
Group D	100 mg/d	20 mg/BIW

(Table 1)

Bayesian optimal interval (BOIN) design method will be used in this clinical trial (Yuan et al,2016; Jin Liu et al., 2018) to determine the maximum tolerated dose (MTD). Given the target DLT rate of 33% for the combination treatment, the BOIN design uses the following optimization rules to make dose fluctuation decisions: if the estimated DLT rate is ≤ 0.26 at the current dose, then escalate to the next higher dose level; if the estimated DLT rate is >0.395 at the current dose, de-escalate to the next lower dose level; otherwise, stay at the current dose level. If the number of patients assigned to single dose reaches twelve and the decision is to stay, then the trial will be stopped. Thus the maximum patient number of each dose level is twelve, or otherwise the trial

will also be terminated; and the maximum total planned sample size is thirty. And whether to stay, escalate or de-esclate will also depends on the available efficacy evidence besides safety.

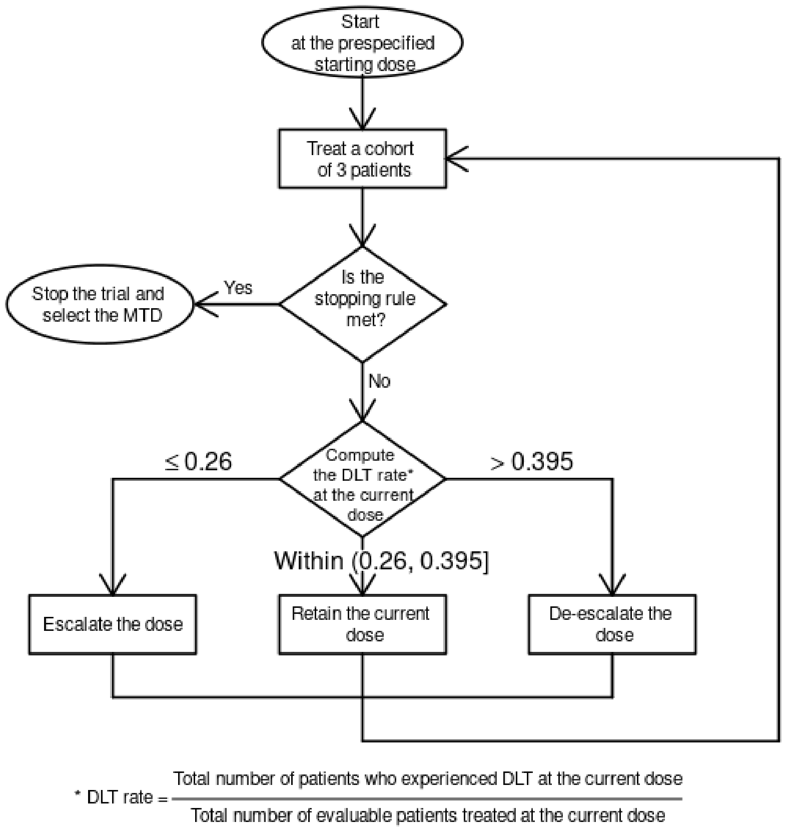

(Figure 1)

Subject Selection and Withdrawal

Inclusion criteria

Subjects must meet all of the following inclusion criteria to be enrolled in this trial:

1. Patients have signed informed consent.

2. Female, aged ≥ 18 years.

3. ECOG performance status: 0-2.

4. Life expectancy ≥ 6 months.

5. Histological or cytological confirmation of estrogen receptor positive and/or

progesterone receptor positive(≥ 10%), HER2-negative locally advanced or metastatic breast cancer not amenable to curative resection or radiotherapy.

6. Prior antitumor therapy: 1) previously received ≤1 line of chemotherapy for recurrent or metastatic breast cancer; 2) Disease recurrence and/or metastasis during or after treatment with Palbociclib or Abemaciclib or Ribociclib in the setting of (neo-)adjuvant therapy, or disease progression during treatment with palbociclib or Abemaciclib or Ribociclib in the metastatic setting ; 3) ≤3 prior lines of endocrine therapy for metastatic disease;

7. At least one extracranial measurable lesion as defined by RECIST v1.1;

8. Adequate organ and bone marrow function:

- Absolute neutrophil count ≥ 1.5 × 10^9/L;
- Platelets ≥ 90 × 10^9/L;
- Hemoglobin ≥ 90g/L;
- Total bilirubin (TBIL) ≤ 1.5 × ULN;
- ALT and AST ≤ 2.5 × ULN;
- Urea/blood urea nitrogen (BUN) and creatinine (Cr) ≤1.5×ULN;
- Left ventricular ejection fraction (LVEF) ≥ 50%;
- The QT correction by the Fridericia formula (QTcF) is < 470 ms.

  INR ≤ 1.5 × ULN, APTT ≤ 1.5 × ULN.

9. Patients recovers from AE related to previous antitumor therapy before the first administration of the study drug (Grade ≤ 1).

Exclusion criteria

Not to be included in any of the following conditions:

1. Previously received treatment with histone deacetylase inhibitor (HDACi);
2. Previously received Dalpiciclib;
3. Leptomeningeal metastasis confirmed by MRI or lumbar puncture;
4. Radiologically confirmed CNS metastases; The following conditions will be excluded: 1) asymptomatic brain metastases not requiring immediate radiotherapy or surgery; 2) Previously received local treatment (radiotherapy or surgery) for

brain metastases, stable for at least 4 weeks, and no symptomatic treatment (including glucocorticoids, mannitol, bevacizumab, etc.) for more than 2 weeks;

5. Patients with visceral crisis (such as lymphangitis carcinomatosis, bone marrow metastasis, leptomeningeal metastasis, diffuse liver metastasis with abnormal liver function), rapid disease progression, and that is not suitable for endocrine therapy;
6. Patients had ascites, pleural effusion and pericardial effusion with clinical symptoms at baseline, which required drainage within 4 weeks before the first medication;
7. Inability to swallow, intestinal obstruction, or other factors that affect medication administration and absorption;
8. Patients who are diagnosed with any other malignancy within 5 years prior to the study, excluding non-melanoma skin cancer treated with radical therapy, basal or squamous cell skin cancer or carcinoma in situ of the cervix and papillary thyroid.
9. Patients has undergone major surgery or major trauma or is expected to undergo major surgery within 4 weeks prior to treatment initiation;
10. Allergy to the any active component or excipient of the investigational drug(s) in this protocol.

Criteria for the termination of treatment

Patients should withdraw/discontinue treatment if any of the following situation occurs during the study:

1. Patients withdrew informed consent and requested withdrawal;
2. Disease progression;
3. Patients who cannot tolerate toxicity;
4. Patients who seriously violated the Protocol and should be terminated as assessed by the investigator;
5. Lost to follow-up or experienced pregnancy events;
6. Other investigator-determined reasons for withdrawal.

Criteria for the termination of study

Study termination criteria include, but are not limited to:

1. Identification of unanticipated, clinically significant, or unacceptable risks to participants;

2. The study drug/trial treatment is ineffective, or continuing the trial is futile;
3. The investigator decided to terminate the study for reasons such as significant delay in recruitment or major protocol violations.

Study Intervention

Study Intervention Administraion

Dalpiciclib: Oral administration, with a 28-day treatment cycle. For the first 3 weeks (Days 1 to 21), the drug is taken continuously, followed by 1 week of rest (Days 22 to 28), during which no medication is administered. It is recommended to take dalpiciclib at approximately the same time each day with lukewarm water, and fasting is required ≥1 hour before and after dosing.

Chidamide: Oral administration, twice a week, with a minimum interval of 3 days between doses (e.g., Monday and Thursday, Tuesday and Friday, Wednesday and Saturday). It should be taken 30 minutes after a meal.

Endocrine Therapy (as chosen by the physician): Refer to the product instructions.

- **Letrozole**: Oral administration, 2.5 mg, once daily, taken on an empty stomach. A 28-day cycle is followed by continuous administration.
- **Fulvestrant:** 500 mg, Intramuscular injection on Day 1 (with an additional dose on Day 15 of the first cycle), administered every 28 days.

Patients will receive treatment according to the study protocol until disease progression (PD), intolerable toxicity, voluntary discontinuation by the patient, or a decision by the physician based on medical events indicating that continuation of the combination therapy is no longer appropriate.

Dose Modifiction

Adverse events associated with dalpiciclib should be managed in accordance with the dose adjustment criteria outlined in Table 2.

Table 2. Dose Modification Guidelines for Dalpiciclib.

Adverse Event	Grade I	Grade II	Grade III	Grade IV
**Hematologic Toxicity**	Maintain the original dose	Maintain the original dose	Discontinue medication, symptomatic treatment, return to Grade I or below. Based on the investigator’s judgment, the original dose may be maintained or reduced by one dose level for the current and subsequent cycles.	Discontinue medication, symptomatic treatment, return to Grade I or below. Based on the investigator’s judgment, the dose should be reduced by one level for the current and subsequent cycles.
**Non-Hematologic Toxicity**	Maintain the original dose	Maintain the original dose or discontinue medication, symptomatic treatment, return to Grade I or below. Based on the investigator’s judgment, the original dose may be maintained or reduced by one dose level for the current and subsequent cycles.	Discontinue medication, symptomatic treatment, return to Grade I or below. Based on the investigator’s judgment, the dose should be reduced by one level for the current and subsequent cycles.	Permanent discontinuation of medication and withdrawal from the study.

Adverse Event	Grade I	Grade II	Grade III	Grade IV
**Febrile Neutropenia**	-	-	Discontinue medication, symptomatic treatment, return to Grade I or below. Based on the investigator's judgment, the dose should be reduced by one level for the current and subsequent cycles.	Permanent discontinuation of medication and withdrawal from the study.

Dose adjustment for Chidamide: Please refer to the prescribing information.

In the event of toxicities clearly related to the investigational drug, management should be determined by the investigator based on the clinical presentation. Treatment may be resumed once the toxicity has resolved to ≤ Grade 1 (or to ≤ Grade 2 if deemed tolerable and without significant safety risk by the investigator). If the same adverse event recurs, management should, in principle, follow the guidelines outlined in Table 2. The investigator may decide to withhold treatment, adjust the dose, or withdraw the patient from the study, prioritizing patient safety to the greatest extent. If the toxicity does not resolve within two weeks after treatment interruption, the patient should, in principle, be withdrawn from the study.

Concomitant medication and concomitant therapy

During the treatment phase, the use of other systemic antitumor therapies or localized antitumor treatments (such as radiotherapy, surgery, etc.) is not permitted. If disease progression, intolerable toxicity, or withdrawal from the study occurs for any

reason (whichever occurs first), alternative antitumor treatment regimens should be selected according to standard clinical practice.

Throughout the study, any comorbidities related to the tumor and other concomitant diseases should be assessed based on routine clinical diagnostic protocols. Appropriate treatment plans should be developed according to the diagnosed conditions.

Treatment for other adverse events should be administered based on clinical guidelines, with detailed collection of relevant treatment information.

Study Procedures

Every effort should be made to ensure the comprehensive collection of efficacy and safety data as required by the study protocol. However, unplanned events may occasionally occur. In such cases, any unplanned assessments conducted for the purpose of evaluating patient safety and treatment efficacy should also be documented and collected. If a protocol-specified assessment cannot be performed, the investigator must record the reason.

All study-related variables will be collected from patient medical records, charts, and patient-reported information. All collected data will be entered into the Case Report Form (CRF) by the investigator. All enrolled patients will be followed from the time of enrollment until the earliest occurrence of study completion, discontinuation of dalpiciclib (as determined by the investigator due to adverse events, toxicity, or other relevant reasons), disease progression, or death.

Study parameters will be collected at baseline and during follow-up. For each patient, the investigator is required to provide the date of signed informed consent. During follow-up visits, the most recent disease assessment date and outcome, as well as dates of disease progression and death (if applicable), should be documented. The variables described in the following sections will be collected to address the study objectives.

Baseline period

- Demographic Data Collection: Gender, date of birth, ethnicity, height, weight, etc.
- ECOG Performance Status
- Vital Signs: Pulse, respiratory rate, body temperature, and blood pressure
- Comprehensive Physical Examination: General condition, head and neck, skin, lymph nodes, eyes, ears, nose, and throat, oral cavity, respiratory system, cardiovascular system, abdomen, genitourinary system, musculoskeletal system, nervous system, and mental status, among others.
- Tumor Diagnosis: Primary and recurrent lesions, date of pathological diagnosis of metastatic lesions, pathological classification, TNM staging, clinical staging, location of metastatic lesions, and the time of disease progression or recurrence after the last treatment. If biopsy of recurrent or metastatic lesions is not feasible, the investigator may discuss with the sponsor and use the pathological results of the primary tumor.
- History of Tumor Treatment
- Surgical History of Tumor: Name of surgery, date of surgery
- Radiation History: Location of radiation, dosage, start and end dates
- Neoadjuvant Treatment History: Treatment regimen, cycles, start and end dates
- Adjuvant Treatment History: Treatment regimen, cycles, start and end dates
- Advanced Treatment History: Treatment regimen, cycles, start and end dates
- Hormonal Therapy History: Medication name, dosage, start and end dates
- History of Comorbidities, Past Medications, and Drug Allergies
- Adverse Events: Adverse events should be recorded starting from the signing of the informed consent form.
- Concomitant Medications/Supportive Treatments: Record concomitant medications/supportive treatments administered within 28 days prior to the first dose of dalpiciclib.
- Pregnancy Testing: For safety reasons, all female patients of childbearing potential must undergo a serum pregnancy test. This test should be conducted within 72 hours prior to the first dose and at the end of treatment visit.

Treatment period

- ECOG Performance Status
- Vital Signs: Pulse, respiratory rate, body temperature, and blood pressure.
- Physical Examination: Targeted physical examinations will be performed as clinically indicated.
- Laboratory Tests: Laboratory parameters will not be routinely collected. If performed, the investigator will assess whether the results are normal or abnormal, and any abnormalities will be recorded in the Case Report Form (CRF).
- Efficacy Assessment: In accordance with the clinical protocols of each center, the investigator will report the date, procedures, and best overall response for each follow-up visit in the CRF, as well as whether the patient benefited from the investigational drug (Yes/No). In cases of disease progression, the physician will assess progression using standard definitions and the institution's standard treatment protocols (e.g., CT scans), documenting the date and recording it in the CRF.
- Subsequent Progression Follow-up: After discontinuation of dalpiciclib due to reasons other than disease progression, patients will continue to be evaluated according to the clinical protocols of each center. The investigator will report the date, procedures, and best overall response at each follow-up visit in the CRF.
- Survival Status: The survival status of the patient (Death, Alive, Unknown) must be recorded in the CRF at each follow-up visit.
- Adverse Events
- Concomitant Medications and Supportive Treatments

Study Assessments

Safety Assessments

Pregnancy Testing

For female patients of childbearing potential, a serum pregnancy test will be conducted prior to the initiation of treatment. Following a negative pregnancy test result during the screening period, appropriate contraception should be implemented. Pregnancy testing will also be routinely performed at the end of treatment or conducted additionally if signs of pregnancy are suspected. If the hCG test is positive, the patient will either discontinue the treatment and exit the study or cease treatment while remaining in the study, depending on the circumstances.

Adverse event

The assessment of adverse events (AEs) includes the type, incidence, severity (graded according to version 5.0 of the NCI CTCAE), onset and resolution times, whether the event is classified as a serious adverse event, its causality, and outcome.

AEs occurring during the study, including signs and symptoms during the screening period, will be recorded on the AE page of the CRF.

Laboratory safety evaluation

Hematological and biochemical tests will be analyzed at local laboratories.

Effectiveness Assessments

High-resolution contrast-enhanced CT or contrast-enhanced MRI is recommended for radiological assessment of tumors. Subjects with a history of contrast agent allergy should be managed according to the contrast allergy prevention guidelines of the respective study center to enable the use of contrast-enhanced CT or MRI whenever

possible. For subjects with absolute contraindications to contrast agents, non-contrast CT scans are permitted.

Screening imaging must include, at a minimum, chest and abdominal scans, and bone scans. Additional imaging of the neck, brain, pelvis, or other regions may be performed based on clinical indications. Investigators may also add scanning regions at baseline or during subsequent tumor assessments as clinically required. Radiologic results obtained within 14 days prior to signing the informed consent, provided they meet study requirements and fall within 28 days before the first dose of study medication, may be used for tumor assessment during the screening period.

Subsequent imaging assessments should be conducted under the same conditions as the baseline scan, including the use of contrast agents. The schedule for tumor imaging assessments will be defined from the start of study treatment and will remain unchanged even in cases of treatment interruption. A ±7-day window is allowed for tumor imaging assessments. The specific time points for these assessments are as follows:

- The first assessment will be performed at the end of the second cycle of treatment; subsequent assessments will occur every two cycles. For patients with baseline bone metastasis, bone scans will be conducted at least once every 8 cycles (approximately 8 months) after the start of treatment.
- For subjects initially assessed as having a complete response (CR) or partial response (PR), confirmation of response must be performed 4 weeks later (before the next scheduled efficacy assessment as per the protocol). Once confirmed, the tumor assessment schedule cannot be altered.
- Tumor assessment will also be conducted at the end of treatment or when a subject discontinues the study (if no tumor assessment has been conducted within the previous 4 weeks).

According to RECIST 1.1 criteria, bone scans and PET scans are not suitable for assessing the efficacy of target lesions. If necessary, these scans may be used to evaluate

non-target lesions, but the frequency of such evaluations can be reduced. For example, bone scans may be repeated only when a target lesion has confirmed CR or when bone disease progression is suspected.

Imaging assessments in this study will be conducted at each study center (on-site review), with evaluations performed by a qualified, experienced investigator designated by the center.

Efficacy assessments based on tumor imaging will be conducted according to the RECIST 1.1 criteria.

The final assessment results will be determined by the investigator's evaluation.

Adverse Events

Defination of Adverse Events

Adverse Events

An adverse event (AE) refers to any unfavorable medical occurrence in a patient enrolled in a clinical trial after signing the informed consent, regardless of whether the event is causally related to the treatment. An AE can include any adverse, unintended symptom, sign, disease, or laboratory abnormality, and must at least encompass the following situations: 1) Pre-existing medical conditions or diseases (present before enrollment in the clinical trial) that are recorded as AEs only if they worsen after the initiation of the investigational drug (including worsening of symptoms, signs, or laboratory abnormalities; 2) Any newly occurring adverse events: Any new medical conditions (including symptoms, signs, or newly diagnosed diseases; 3) Abnormal but clinically significant laboratory findings.

Diagnostic or therapeutic invasive procedures (such as surgery) or non-invasive interventions should not be reported as AEs. However, if the disease condition leading to the procedure meets the definition of an AE, it should be reported as such. For

example, acute appendicitis occurring during the AE reporting period should be recorded as an AE, while the subsequent appendectomy should be documented as the treatment for that AE.

Serious Adverse Events

A serious adverse event (SAE) is an adverse event that occurs during a clinical trial and meets one or more of the following criteria:

- An event that results in death;
- An event that is life-threatening (the term "life-threatening" refers to an event/reaction during which the patient is at immediate risk of death, but does not imply that the condition worsening may lead to death);
- An event that requires hospitalization or prolongs an existing hospitalization;
- An event that results in permanent or significant disability or loss of function;
- A congenital anomaly or birth defect;
- Other medically significant events: These are adverse events/reactions that, although not immediately life-threatening, fatal, or requiring hospitalization, may pose a significant risk to the patient based on sound medical and scientific judgment, or may require intervention (such as medication or surgery) to prevent the serious consequences listed above.

Classification of Adverse Events

Ralationship of Adverse Events

The investigator should conduct a comprehensive assessment to determine whether there is a reasonable possibility that the investigational drug caused or contributed to the adverse event (AE). The factors to consider include whether the occurrence of the AE follows a reasonable temporal sequence after the administration of the investigational drug, the characteristics of the investigational drug, its toxicological and pharmacological effects, concomitant medication use, the patient's

underlying conditions, medical history, family history, and any potential for triggering or reactivating responses. The investigator should then evaluate the potential relationship between the AE and the investigational drug using a five-level classification: “Definite, Probably, Possibly, Probably Not, or Unassessable.”

Severity of Adverse Events

Refer to the grading criteria for drug AE in NCI-CTC AE 5.0. If AEs not listed in the NCI-CTC AE 5.0 table, refer to the criteria below:

Table 3 Reference criteria for severity of AEs not listed in NCI-CTC AE 5.0

Grade	Clinical description of the severity level
1	Mild; asymptomatic or mild; clinical or diagnostic observation only; treatment-free.
2	Moderate; requiring small, local or non-invasive treatment; referring to cooking, clothing purchase, telephone use, financial management, etc.
3	Serious or medically significant but not immediately threatening life; hospitalization or prolonged hospitalization; disability; restricted activities of daily life. Self-rational daily living activities refer to bathing, dressing and eating<br>Rice, toilet, medicine, etc., are not bedridden.
4	Life-threatening; requiring urgent treatment
5	Death associated with an AE

Collection and Follow-up of Adverse Events

Collection of AE / SAE should start when the subject signed the informed consent until the end of the safety follow-up period.

All AEs / SAEs should be followed until the end or disappearance of the safe follow-up period, remission to baseline or level 1, stable status, or reasonably explained (e. g. lost to follow-up, death).

The investigator should inquire about the AE / SAEs occurring after the last visit at each visit and provide timely follow-up information based on the requests for further evaluation.

Reporting Serious Adverse Events

In the event of a serious adverse event (SAE), whether it is a first-time or follow-up report, the investigator must promptly complete the Serious Adverse Event (SAE) Report Form, sign it, and date it. The SAE must be reported to the sponsor and relevant departments within 24 hours of the investigator becoming aware of the event.

All serious adverse events (SAEs) should be thoroughly documented, including symptoms, severity (according to NCI-CTCAE version 5.0), the relationship with each investigational drug, the timing of onset, the timing of interventions, actions taken with respect to each investigational drug due to the SAE, follow-up duration and methods, as well as the outcome. If the investigator believes that a serious adverse event is unrelated to the investigational drug but may be potentially associated with study conditions (e.g., discontinuation of prior treatment or comorbidities during the trial), this relationship should be clearly detailed in the narrative section of the SAE report in the case report form. If the intensity of an ongoing SAE or its relationship with the investigational drug changes, a follow-up report should be submitted immediately. If the investigator believes that there was an erroneous report of a previously submitted SAE, corrections, retractions, or downgrading should be made in the follow-up report and submitted according to the SAE reporting procedures.

Pregnancy

If the female subjects were pregnant during the clinical study, the subjects must immediately discontinue the investigational drug and withdraw from the study.

During the clinical study, the investigator must complete the Clinical Trial Pregnancy Report/Follow-up Form and report the pregnancy event to the relevant departments within 24 hours of becoming aware of the event.

The investigator is required to track the pregnancy event until the final outcome (including any pregnancy termination or delivery). Follow-up for delivery should

continue until 1 month postpartum, with the results reported to both the relevant departments and HengRui Drug Safety Department.

If the pregnancy result is an ectopic pregnancy, spontaneous abortion, intrauterine fetal death, neonatal death, or congenital abnormalities, it should be considered an SAE and reported in accordance with the SAE reporting timelines.

If a subject has an SAE during pregnancy, complete the Serious Adverse Event Report Form and report with SAE reporting time and procedures.

Disease Progression and Death

Disease progression was defined as the deterioration of the subject's condition due to the indication of the study drug. Including radiographic evidence of progression, or the progression of clinical symptoms and signs. The new metastasis of the primary tumor, or the progression of the original metastasis were considered to be disease progression. Life-threatening events resulting from symptoms and signs of disease progression, requiring hospitalization or prolonged hospitalization, or causing permanent or severe disability / dysfunction / affecting work capacity, congenital abnormalities or birth defects, are not reported as SAEs. If there is any uncertainty about whether the SAE is due to disease progression, it should be reported as an SAE.

In the study population of this trial, "disease progression" is expected to occur and should not be reported as an AE term. When disease progression occurs, the event used to confirm disease progression should be reported as an AE. For example, the subject developed epilepsy and was determined to be related to brain metastasis, and the AE term should be recorded as "epilepsy" rather than "disease progression" or "brain metastasis".

If the subject dies during the trial, whether he or she has received new antitumor therapy, it must be reported as a SAE.

Death as assessed by the investigator due to signs and symptoms of disease progression may be reported as an SAE. However, the word "death" should not be used

as an AE or a SAE term, but as the result of an event. The event causing or leading to death should be recorded as an SAE. If the cause of death is unknown and could not be determined at the time of reporting, the SAE term is recorded as "unexplained death".

Statistical Analysis Methods

Sample Size Determination

Bayesian optimal interval (BOIN) design will be utilized to determine the maximum toxicity dose, with a maximum of twelve subjects enrolled in each dose group and a maximum of thirty subjects enrolled in the entire study.

The BOIN design uses the following optimization rules to make dose fluctuation decisions: if the estimated DLT rate is ≤ 0.26 at the current dose, then escalate to the next higher dose level; if the estimated DLT rate is >0.395 at the current dose, then de-escalate to the next lower dose level; otherwise, remain at the current dose level.

Analysis Population

The analysis population of this study included the full analysis set (Full Analysis Set, FAS), the per-protocol set (Per Protocol Set, PPS), and the safety set (Safety Set, SS).

Full Analysis Set (FAS) will include all subjects who received at least one dose of the study treatment.

The Safety Analysis Set (SS) will include all subjects who received at least one dose of the study treatment. This dataset will be used for safety analysis.

The Per-protocol Analysis Set (PPS) will include all subjects who received at least 1 dose of study treatment and without any major protocol deviation which may have impact on the efficacy.

Statistical Analyses

General Methods

Unless otherwise stated, all data will be analyzed using appropriate statistics according to the data type: for the continuous data, mean, standard deviation, median and range (minimum, maximum) will be calculated; for counting data, frequency and the corresponding percentages will be summarized, given as n (%). The Kaplan–Meier method will be used for analyzing time-to-event data to estimate the median survival time, and the corresponding 95% confidence intervals will be calculated based on Brookmeyer-Crowley method. If necessary, Kaplan-Meier curves will be also presented.

Subject Disposition

Subject disposition information, including but not limited to the number of subjects being enrolled, treated, withdrawing from the trial, and major protocol deviations occurring in patients, will be summarized. The reasons for screening failure and the reasons for withdrawal from the trial will also be summarized.

Demographics and baseline characteristics

The demographic characteristics including age(years), hormone receptor status, genetic mutation status and so on, as well as other baseline clinical characteristics including tumor history, prior therapies will be summarized by descriptive statistics.

Safety Analysis

In this study, the primary endpoint of maximum tolerated dose (MTD) will be determined utilizing BOIN design. Dose-limiting toxicities (DLT) will be summarized in each dose group and SS population. The number and percentage of subjects experienced DLT(s) will be summarized.

The incidence of AEs, AEs of grade ≥3, AEs that lead to dosage adjustments, AEs that lead to termination of therapy will be summarized for each intervention group. They will be further summarized by preferred term (PT) and severity. If applicable, the Kaplan–Meier method will be used to analyze the time to AE occurrence and duration of AE for AEs of special concern.

Efficacy Analyses (Secondary)

The ORR and DCR will be analyzed for FAS population and for each dose group, and the corresponding 95% confidence interval (CIs) of will be calculated using the Clopper–Pearson method based on binomial distribution.

For progression-free survival (PFS), Kaplan–Meier method will be adopted to analyzed median PFS (mPFS), and theBrookmeyer–Crowley method will be used to estimate the corresponding 95% confidence intervals. The Kaplan-Meier curves will also be presented. Subgroup analysis will be performed for duration of prior CDK4/6 inhibitor treatment, prior therapy lines and genetic mutation status, et al.

Research management

Compliance with Ethical and Regulatory Guidelines

This clinical trial must comply with the Declaration of Helsinki, the Good Laboratory Practice (GCP) of the NMPA (formerly CFDA) and relevant regulations.

Institutional and Ethics Norms

Before the study, approval from the Ethics committee must be obtained. During the clinical study, any amendments to the study protocol must be submitted to the Ethics Committee. The investigator has the responsibility to submit the mid-term report

regularly according to the relevant requirements of the EC, and shall notify the EC that the trial has ended.

Informed Consent

The subject must give informed consent to participate in the trial before receiving the drug to protect the legitimate rights and interests of the subject. The investigator has the responsibility to fully and comprehensively inform the subject or his designated representative about the purpose of the study, the role of the drug, possible toxic effects and possible risks, informing the subject of their rights, risks and benefits. Talking is an extremely important informed consent process. If the subject and his legal representative are not literate, the informed consent process shall be attended by the witness. After the oral consent of the subject or the legal representative signs the informed consent form, and the signature of the witness shall be on the same day as that of the signature of the subject. The informed consent form shall indicate the version number and version date.

Privacy

Investigators must ensure that subjects' privacy is not disclosed to any unauthorized third parties, and documents such as the informed consent form must be kept strictly confidential.

Quality Assurance and Control

During the course of the test, periodic inspection visits will be made to the test center to ensure that the test plan is carried out. Review primary data to confirm consistency of data on the case report form.

Data Management

Data Collection and Quality Control

This study uses the CRF for data collection. The investigator or dedicated data entry personnel (CRC) shall enter the data into the data collection system in accordance with the requirements of the visit process and the CRF filling guidelines. The investigator or data entry personnel are permitted to modify or clarify problematic data, and any correction can only be underlined, annotated by margins, and dated with the signature of the investigator. Monitors, medical reviewers, and other relevant personnel will also review the CRF data as needed and raise queries regarding any questionable data. Investigators are expected to respond promptly to queries raised by the data reviewers.

Data Storage

To ensure the evaluation and supervision of clinical research by the National Medical Products Administration (NMPA), investigators must agree to retain all study-related documents, including original records of patient hospitalizations, informed consent forms, case report forms (CRFs), detailed records of drug distribution, and other relevant materials. These study documents must be stored by the research institution for a period of 5 years after the completion of the clinical trial. Ownership of all materials related to this clinical study belongs to the sponsor, and unless required by the NMPA, no document shall be provided to any third party in any form without the written consent of the sponsor. The storage and management of study data must comply with Good Clinical Practice (GCP) requirements, and essential clinical study documents must be retained for 5 years after the termination of the clinical research.

Protocal Revision

No one can modify the protocol except the study leader. Any necessary changes to the protocol shall be made in the form of protocol revision, and shall be submitted to the Ethics Committee for approval or filing after signing and agreeing by the investigator, and the details of previous modifications shall be explained in the protocol.

Reference

[1]. Spring LM, Wander SA, Zangardi M, et al.. CDK 4/6 inhibitors in breast cancer: current controversies and future directions. Curr Oncol Rep 2019;21:25.

[2]. Finn RS, Martin M, Rugo HS, et al.. Palbociclib and letrozole in advanced breast cancer. N Engl J Med 2016;375:1925–1936.

[3]. Cristofanilli M, Turner NC, Bondarenko I, et al.. Fulvestrant plus palbociclib versus fulvestrant plus placebo for treatment of hormone-receptor-positive, HER2-negative metastatic breast cancer that progressed on previous endocrine therapy (PALOMA-3): final analysis of the multicentre, double-blind, phase 3 randomised controlled trial. Lancet Oncol 2016;17:425–439.

[4]. Hortobagyi GN, Stemmer SM, Burris HA, et al.. Ribociclib as first-line therapy for HR-positive, advanced breast cancer. N Engl J Med 2016;375:1738–1748.

[5]. Goetz MP, Toi M, Campone M, et al.. MONARCH 3: abemaciclib as initial therapy for advanced breast cancer. J Clin Oncol 2017;35:3638–3646.

[6]. Sledge GW Jr, Toi M, Neven P, et al.. MONARCH 2: abemaciclib in combination with fulvestrant in women with HR+/HER2– advanced breast cancer who had progressed while receiving endocrine therapy. J Clin Oncol 2017;35:2875–2884.

[7]. Herrera-Abreu MT, Palafox M, Asghar U, et al.. Early adaptation and acquired resistance to CDK4/6 inhibition in estrogen receptor-positive breast cancer. Cancer Res 2016;76:2301–2313.

[8]. Yang C, Li Z, Bhatt T, et al.. Acquired CDK6 amplification promotes breast cancer resistance to CDK4/6 inhibitors and loss of ER signaling and dependence. Oncogene 2017;36:2255–2264.

[9]. Formisano L, Lu Y, Servetto A, et al.. Aberrant FGFR signaling mediates resistance to CDK4/6 inhibitors in ER+ breast cancer. Nat Commun 2019;10:1373.

[10]. Caldon CE, Sergio CM, Kang J, et al.. Cyclin E2 overexpression is associated with endocrine resistance but not insensitivity to CDK2 inhibition in human breast cancer cells. Mol Cancer Ther 2012;11:1488–1499.

[11]. Drago JZ, Formisano L, Juric D, et al.. FGFR1 gene amplification mediates endocrine

resistance but retains TORC sensitivity in metastatic hormone receptor positive (HR+) breast cancer. Clin Cancer Res 2019;25:6443–6451.

[12]. P. Zhang, Q.Y. Zhang, X. Hu, et al. Dalpiciclib plus fulvestrant in HR+/HER2− advanced breast cancer (ABC): Updated analysis from the phase III DAWNA-1 trial. 2022 ESMO, 229P.

[13]. Binghe Xu, Qingyuan Zhang, Pin Zhang, et al. Dalpiciclib plus letrozole or anastrozole as first-line treatment for HR+/HER2- advanced breast cancer (DAWNA-2): a phase 3 trial. 2022 ESMO, LBA16.

[14]. Hamilton E, Infante JR. Targeting CDK4/6 in patients with cancer. Cancer Treat Rev. 2016 Apr;45:129-38. doi: 10.1016/j.ctrv.2016.03.002. Epub 2016 Mar 8. PMID: 27017286.

[15]. Zhou M, Yuan M, Zhang M, Lei C, Aras O, Zhang X, An F. Combining histone deacetylase inhibitors (HDACis) with other therapies for cancer therapy. Eur J Med Chem. 2021 Dec 15;226:113825. doi: 10.1016/j.ejmech.2021.113825. Epub 2021 Sep 4. PMID: 34562854; PMCID: PMC9363153.

[16]. Xue J, Wu G, Ejaz U, Akhtar F, Wan X, Zhu Y, Geng A, Chen Y, He S. A novel histone deacetylase inhibitor LT-548-133-1 induces apoptosis by inhibiting HDAC and interfering with microtubule assembly in MCF-7 cells. Invest New Drugs. 2021 Oct;39(5):1222-1231. doi: 10.1007/s10637-021-01102-9. Epub 2021 Mar 31. PMID: 33788074.

[17]. Jiang Z, Li W, Hu X, et al. Tucidinostat plus exemestane for postmenopausal patients with advanced, hormone receptor-positive breast cancer (ACE): a randomised, double-blind, placebo-controlled, phase 3 trial[J]. The Lancet Oncology, 2019, 20(6): 806-815.

Exploration of Dalpiciclib + Chidamide in HR+/HER2- advanced breast cancer after failure of CDK4/6 inhibitor: a phase Ib study

Statistical Analysis Plan (SAP)

Site in Charge: The Fifth Medical Center of the Chinese People's Liberation Army General Hospital

Principal investigator: Prof. Tao Wang

ClinicalTrials.gov Identifier: NCT 05586841

TABLE OF CONTENTS

LIST OF ABBREVIATIONS AND DEFINITIONS OF TERMS 59
1. INTRODUCTION 60
2. STUDY OBJECTIVES 61
2.1. Objectives 61
3. STUDY DESIGN AND METHODS 62
3.1. General Study Design and Plan 62
3.2. Randomization 63
3.3. Blinding 64
4. STUDY ENDPOINTS 65
4.1. Primary Endpoint(s) 65
4.2. Secondary Endpoint(s) 65
4.3. Health Economics and Outcome Research Endpoint(s) 65
5. SAMPLE SIZE DETERMINATION 66
6. GENERAL AND STATISTICAL CONSIDERATIONS 67
6.1. Analysis Sets 67
6.1.1. Full Analysis Set 67
6.1.2. Safety Analysis Set 67
6.1.3. Per-Protocol Analysis Set 67
6.2. General Considerations 67
6.2.1. Reference Start Date, End Date and Study Day 67
6.2.2. Baseline 67
6.2.3. Definition and Use of Visit Windows 67
6.2.4. Repeated or Unscheduled Assessments of Safety Parameters 67
6.3. Statistical Considerations 67
6.3.1. Missing Data 67
6.3.2. Character Values of Clinical Laboratory Tests 69
6.3.3. Computing Methods and Reporting Conventions 69
6.3.4. Subgroups 70
7. STATISTICAL ANALYSIS 71
7.1. Summary of Study Data 71

7.1.1. Subject Disposition 71
7.1.2. Protocol Deviations 71
7.1.3. Demographic and Baseline Characteristics 71
7.2. Safety Analyses 72
7.2.1. Dose-limiting Toxicities 72
7.2.2. Adverse Events 72
7.2.3. Clinical Laboratory Evaluations 72
7.2.4. Vital Sign 72
7.3. Efficacy Analyses 72
8 SUMMARY OF CHANGES TO THE STATISTICAL ANALYSES SPECIFIED IN PROTOCOL 73
9 APPENDIX 73

LIST OF ABBREVIATIONS AND DEFINITIONS OF TERMS

ABBREVIATION	DEFINITION
AE	Adverse event
BOIN	Bayesian optimal interval design
CTCAE	Common terminology criteria for adverse event
DLT	Dose-limiting toxicity
ECOG	Eastern cooperative oncology group
ECG	Electrocardiogram
FAS	Full analysis set
MTD	Maximum tolerated dose
MedDRA	Medical Dictionary for Regulatory Activities
PFS	Progression-free survival
PS	Performance status
PT	Preferred term
RECIST	Response Evaluation Criteria in Solid Tumors
SAP	Statistical analysis plan
SAS	Statistical Analysis System
SS	Safety analysis set

INTRODUCTION

This statistical analysis plan (SAP) serves as a guide mainly for the analysis of the safety and efficacy endpoints. The SAP provides a detailed, technical elaboration of the statistical analyses of safety and efficacy data as described in the study protocol Version 1.0 dated August 28, 2022.

STUDY OBJECTIVES

Objectives

- To evaluate the safety and tolerability of the combination of an HDAC inhibitor (Chidamide), a CDK4/6 inhibitor (dalpiciclib) and endocrine therapy in patients with HR+/HER2- advanced breast cancer who have progressed after prior CDK4/6 inhibitor treatment. Based on toxicity and efficacy outcomes observed in participants, four dose levels will be assessed and the optimal dose will be determined.
- To assess the efficacy and safety of dalpiciclib in combination with Chidamide and endocrine therapy in patients with HR+/HER2- advanced breast cancer who have progressed after prior CDK4/6 inhibitor treatment.

STUDY DESIGN AND METHODS

General Study Design and Plan

This is a phase 1B study to evaluate the safety and tolerability of dalpiciclib in combination with chidamide in patients with HR+/HER2- advanced breast cancer after the failure of CDK4/6 inhibitor therapy. To explore the maximum tolerated dose(MTD), Bayesian optimal interval (BOIN) design will be used, with a maximum of twelve subjects enrolled in each dose group and a maximum of thirty subjects enrolled in the entire study.

Dalpiciclib will be administered in a dose of 100 mg/d or 125 mg/d and chidamide shall be administered in a dose of 25 mg/BIW or 20 mg/BIW. Four dose levels will be established as follows:

Dose group	Dalpiciclib	Chidamide
Group A	125 mg/d	25 mg/BIW
Group B	125 mg/d	20 mg/BIW
Group C	100 mg/d	25 mg/BIW
Group D	100 mg/d	20 g/BIW

Bayesian optimal interval (BOIN) design method will be used in this clinical trial (Yuan et al,2016; Jin Liu et al., 2018) to determine the maximum tolerated dose (MTD). Given the target DLT rate of 33% for the combination treatment, the BOIN design uses the following optimization rules to make dose fluctuation decisions: if the estimated DLT rate is ≤ 0.26 at the current dose, then escalate to the next higher dose level; if the estimated DLT rate is >0.395 at the current dose, de-escalate to the next lower dose level; otherwise, stay at the current dose level. If the number of patients assigned to single dose reaches twelve and the decision is to stay, then the trial will be stopped. Thus the maximum patient number of each dose level is twelve, or otherwise the trial will also be terminated; and the maximum total planned sample size is thirty. And whether to stay, escalate or de-esclate will also depends on the available efficacy evidence besides safety.

DLT will be defined as the following drug-related or potentially drug-related adverse events occurring within the first cycle per CTC-AE V5.0, which are elaborated in detail in the protocol:

3. Hematological toxicity

- Grade 4 neutropenia lasted for ≥5 days;
- Grade 4 thrombocytopenia, or grade 3 thrombocytopenia with clinically significant bleeding;
- Grade ≥ 3 neutropenia with fever (≥ 38.0 ℃ for 1 hour or > 38.3 ℃);
- Grade ≥ 4 anemia.

4. Non-hematological toxicity

   Any grade ≥ 3 non-hematological toxicity, except:

   - Grade 3-4 nausea/vomiting and/or diarrhea and/or electrolyte disturbances that have recovered to grade ≤ 2 within 72 hours after optimal supportive treatment;
   - There will be a definite increase of grade 3-4 alkaline phosphatase and glutamyl transpeptidase related to tumor but not related to treatment.

All the subjects will receive the regimen described as below:

Dalpiciclib: Oral administration, with a 28-day treatment cycle. For the first 3 weeks (Days 1 to 21), the drug is taken continuously, followed by 1 week of rest (Days 22 to 28), during which no medication is administered. It is recommended to take dalpiciclib at approximately the same time each day with lukewarm water, and fasting is required ⩾1 hour before and after dosing.

Chidamide: Oral administration, twice a week, with a minimum interval of 3 days between doses (e.g., Monday and Thursday, Tuesday and Friday, Wednesday and Saturday). It should be taken 30 minutes after a meal.

Endocrine Therapy (as chosen by the physician): Refer to the product instructions.

- Letrozole: Oral administration, 2.5 mg, once daily, taken on an empty stomach. A 28-day cycle is followed by continuous administration.

- Fulvestrant: 500 mg, Intramuscular injection on Day 1 (with an additional dose on Day 15 of the first cycle), administered every 28 days.

Patients will receive treatment according to the study protocol until disease progression (PD), intolerable toxicity, voluntary discontinuation by the patient, or a decision by the physician based on medical events indicating that continuation of the combination therapy is no longer appropriate.

Randomization

Not applicable.

Blinding

Not applicable.

STUDY ENDPOINTS

Primary Endpoint(s)

Maximum tolerated dose (MTD) is defined as the highest dose level at which no more than one-third of subjects experiencing dose-limiting toxicities (DLT) during the completion of one treatment cycle.

Secondary Endpoint(s)

- Objective response rate (ORR): defined as the proportion of subjects whose best overall response (BOR) is CR or PR, evaluated according to RECIST v1.1.
- Disease control rate (DCR): defined as the proportion of subjects whose best overall response (BOR) is CR, PR or stable disease, evaluated according to RECIST v1.1.
- Progression-free survival (PFS): defined as the time from the first dose of any treatment drug to the date of documented disease progression or death due to any cause, whichever occurs earlier.
- Incidence of adverse events

Health Economics and Outcome Research Endpoint(s)

- Not applicable.

SAMPLE SIZE DETERMINATION

Bayesian optimal interval (BOIN) design will be utilized to determine the maximum toxicity dose, with a maximum of twelve subjects enrolled in each dose group and a maximum of thirty subjects enrolled in the entire study.
The BOIN design uses the following optimization rules to make dose fluctuation decisions: if the estimated DLT rate is ≤ 0.26 at the current dose, then escalate to the next higher dose level; if the estimated DLT rate is >0.395 at the current dose, then de-escalate to the next lower dose level; otherwise, remain at the current dose level.

GENERAL AND STATISTICAL CONSIDERATIONS

Analysis Sets

1.1.1. Full Analysis Set

The Full Analysis Set (FAS) will include all subjects who received at least one dose of the study treatment.

1.1.2. Safety Analysis Set

The Safety Analysis Set (SS) will include all subjects who received at least one dose of the study treatment. This dataset is used for safety analysis.

1.1.3. Per-Protocol Analysis Set

The Per-protocol Analysis Set (PPS) will include all subjects who received at least 1 dose of study treatment and without any major protocol deviation which may have impact on the efficacy. The list of major protocol deviations and subjects who may need to be excluded from PPS should be finalized before data base lock.

General Considerations

1.1.4. Reference Start Date, End Date and Study Day

- Reference start date is the first date of study treatment administration
- Reference end date is the last date of study treatment administration.

Study day will be calculated based on the reference start date:

- Study day = event/assessment date – reference start date + (event/assessment date ≥ reference start date).

1.1.5. Baseline

In general, baseline for is defined as the last assessment before first date of study drug administration, unless otherwise specified.

1.1.6. Definition and Use of Visit Windows

All the by-visit analysis will use the original visits being recorded in the CRF. No derivation of visit will be performed.

1.1.7. Repeated or Unscheduled Assessments of Safety Parameters

In general, all the by-visit summaries will only present the data being recorded in scheduled visits. Measurements recorded in unscheduled visits will not be included in the by-visit summaries, but will contribute to determine the best/worst case value if required (i.e. shift table). If there are multiple values for the same test before either the initiation of study treatment, the last available assessment will be used.

Statistical Considerations

1.1.8. Missing Data

Missing Date or Incomplete Date

In general, the incomplete date will be imputed following the below rules unless otherwise specified if it has impact on the statistical analysis:

- Impute 15 to the day part if only day is missing;
- Impute JUL 01 to the month and day if both month and day is missing;
- Will not impute if completely missing.

Missing or Incomplete Date Information of Study Treatment

In general, the date of study treatment should not be imputed. If date of study treatment is missing or partial missing, all the efforts should be made to obtain the date.

If the last date of study treatment administration is missing due to data cut-off, the date should be imputed to the cut-off date.

Incomplete Date Information for Prior and Concomitant Medications

The date of concomitant medication will only be imputed when partial missing.

Incomplete Start Dates

- Missing Day and/or Month: impute to the earliest possible date.
- Complete missing date will not be imputed and will be considered as concomitant medication.

In case the imputed start date is after the stop date, then the start date will be imputed using the stop date.

1.1.8.1.2. Incomplete Stop Dates

- Month and day are missing: impute to Dec 31.
- Only day is missing: impute to the last day of the month
- Only month is missing: impute to Dec.

The imputed stop date will then be compared with last date known to be alive or the death date. If the imputed date is later than the reference date, then impute to the date being referenced.

1.1.8.4. Incomplete Date Information for Adverse Events

6.3.1.4.1 Incomplete Start Dates

- Missing Day and Month
    - If the year of the incomplete date is the same as the year of the first date of study treatment administration, then impute to the date of first study treatment administration.
    - If the year of the incomplete date is prior to the year of the first date of study treatment administration, then impute to the Dec 31.

- If the year of the incomplete date is after the year of the first date of study treatment administration, then impute to Jan 1.

- Missing Day Only
  - If the known parts of the incomplete date are the same as the corresponding parts of the first date of study treatment administration, then impute to the date of first study treatment administration.
  - If the known parts of the incomplete date is prior to the corresponding parts of the first date of study treatment administration, then impute to the last day of the month.
  - If the known part of the incomplete date is after the year of the first date of study treatment administration, then impute to the first day of the month.

In case the imputed start date is after the stop date, then the start date will be imputed using the stop date.

6.3.1.4.2 Incomplete Stop Dates

- If completely missing or the year is missing for the adverse events whose status are not ongoing: impute to the date of last date known to be alive or death date;
- If month and/or day is missing: impute to the latest possible date.

1.1.8.5. Incomplete death date

- If the only the day is missing: impute to the first day of the month
- If both the month and day are missing: impute with Jan 01
- If completely missing: impute with the next day of last date known to be alive.

The imputed date should be later than the last date known to be alive.

1.1.9. Character Values of Clinical Laboratory Tests

If the reported value for a clinical laboratory test is in form of a character string, then the numeric part of the string will be used for the analysis purpose.

If the result is reported as below-limit-of quantification (BLQ), then the lower boundary of the quantifiable assessment range will be used for the analysis purpose. However, the actual values as reported in the database will be presented in data listings.

1.1.10. Computing Methods and Reporting Conventions

All the statistical analysis will be performed with SAS® Version 9.4 or above.

Statistical Summary Conventions

For continuous variables, descriptive statistics including number of subjects with non-missing values, mean, standard deviation, median, minimum, and maximum, will be presented. For categorical variables, frequencies and percentages will be presented.

Time-to-events data will be analyzed by Kaplan-Meier method, and K-M plots will be presented if necessary.

General Reporting Conventions

The means and medians should have 1 more decimal place than the observed values, the standard deviations should have 2 additional decimal places than the observed values. Min and Max should have the same decimal place with the observed values.

1.1.11. Subgroups

Subgroup analysis will be conducted for PFS. It should be noted that the study is not designed to detect the difference in treatment within subgroups and the type-I error rate is not controlled for the subgroup analysis.

The following subgroups will be assessed for PFS:

- Prior CDK4/6 inhibitor treatment duration (≤12 months, >12 months)
- Prior therapy lines (≤ 3 prior therapy lines, > 3 prior therapy lines)
- TP53 mutation (mutated, wild type)
- PIK3CA mutation (mutated, wild type)
- ESR1 mutation (mutated, wild type)

STATISTICAL ANALYSIS

Summary of Study Data

1.1.12. Subject Disposition

The number of subjects being screened will be summarized, the number and percentages of subjects who failed screening as well as reasons of screen failure will also be summarized. The number and percentage of subjects received treatment, completed/not completed treatment and still on study treatment/discontinued treatment will be summarized by dose group.

The number and percentage of subject in each analysis set (Full analysis set, safety analysis set and per-protocol analysis set) will be summarized.

In addition, the duration of subject in study will also be summarized:

- Duration in study (months) = (Last day of known to be alive – first treatment date + 1)/30.4375

1.1.13. Protocol Deviations

Protocol deviations will be summarized and further summarized by severity. Major protocol deviation leading to the exclusion of subjects from PPS will also be summarized. Protocol deviations will be listed, and the major protocol deviations will be flagged.

1.1.14. Demographic and Baseline Characteristics

Statistical description will be performed on demographic and baseline characteristics for FAS population. The demographic characteristics and diagnosis information listed below will be summarized.

- Age (median, range)
- Gender (male, female)
- Baseline ECOG PS (0, 1)
- Hormone receptor (ER+/PR+, ER+/PR−)
- Number of metastatic sites (≥3, <3)
- Metastatic sites
- Prior CDK4/6 inhibitor treatment duration (≤12 months, >12 months)
- Prior therapy lines (≤ 3 prior therapy lines, > 3 prior therapy lines)
- Prior therapies

Safety Analyses

All the DLT related analysis will be performed in SS population. All the general safety analysis will be performed for SS population except for otherwise specified.

1.1.15. Dose-limiting Toxicities

Dose-limiting toxicities will be summarized in SS population. The number and percentage of subjects experienced DLT(s) will be summarized. Furthermore, DLT(s) will be summarized by preferred term and NCI-CTCAE grade.

1.1.16. Adverse Events

All adverse events will be coded by low-level term, preferred term and major system organ class (SOC), etc. using MedDRA 25.0 or above. and graded according to NCI-CTCAE 5.0. Under the same System Organ Class (SOC) and/or Preferred Term (PT).

Treatment-emergent adverse events are defined as any AEs that occur after starting the use of the trial drug. The incidence of AEs, SAEs, AEs of grade ≥3, treatment-related AEs, drug-related SAEs will be summarized for each group. They will be further summarized by preferred term (PT) and severity. If applicable, the Kaplan–Meier method will be used to analyze the time to AE occurrence and duration of AE for AEs of special concern.

1.1.17. Clinical Laboratory Evaluations

The abnormality for each laboratory parameter will be assessed by investigator. A shift table from baseline to the worst post-baseline assessment according to the investigator's evaluation for each parameter listed below will be presented.

1.1.18. Vital Sign

The results and their changes from baseline of each vital sign parameter will be summarized by descriptive statistics for baseline (changes from baseline is not applicable) and each post-baseline scheduled visits.

Data listing will be provided for the vital sign results.

Efficacy Analyses

The objective response rate (ORR) and disease control rate (DCR) of will be analyzed for each group and all subjects in FAS population and for each group. The 95% confidence intervals (CI) will be calculated using the Clopper–Pearson method based on binomial distribution.

The Kaplan-Meier method will be used to analyzed PFS for each group and all subjects in FAS and the corresponding 95% CIs will be calculated by Brookmeyer-Crowley method. The Kaplan-Meier curves will also be presented. In addition, the PFS rates at 12 and 24 months as well as corresponding 95% confidence intervals will be presented.

8 SUMMARY OF CHANGES TO THE STATISTICAL ANALYSES SPECIFIED IN PROTOCOL

Not applicable.

9 APPENDIX

Dose Escalation, De-Escalation, and Elimination Boundaries

Action	The Number of Subjects Treated at the Current Dose																											
	3	4	5	6	7	8	9	10	11	12	13	14	15	16	17	18	19	20	21	22	23	24	25	26	27	28	29	30
Escalate if # of DLT	0	1	1	1	1	2	2	2	2	3	3	3	3	4	4	4	4	5	5	5	5	6	6	6	7	7	7	7
Stay if # of DLT=	1	NA	NA	2	2	3	3	3	3-4	4	4-5	4-5	4-5	5-6	5-6	5-7	5-7	6-7	6-8	6-8	6-9	7-9	7-9	7-10	8-10	8-11	8-11	8-11
De-escalate if # of DLT >=	2	2	2	3	3	4	4	4	5	5	6	6	6	7	7	8	8	8	9	9	10	10	10	11	11	12	12	12
Eliminate if # of DLT >=	3	3	4	4	5	5	6	6	7	7	8	8	8	9	9	10	10	11	11	11	12	12	13	13	13	14	14	15

DLT = dose-limiting toxicity; NA = not applicable.
