# Supplementary material for: Dalpiciclib plus chidamide in HR + /HER2−advanced breast cancer after CDK4/6 inhibitor failure: a phase Ib trial
Source: Nat Commun. 2026 Mar 20;17:5177. doi: 10.1038/s41467-026-70650-6 (PMC13254248; doi:10.1038/s41467-026-70650-6)
Supplement: Supplementary file 2 — Reporting Summary [file 41467_2026_70650_MOESM2_ESM.pdf]

nature portfolio

Corresponding author(s): Tao Wang

Last updated by author(s): Jan 4, 2026

Reporting Summary

Nature Portfolio wishes to improve the reproducibility of the work that we publish. This form provides structure for consistency and transparency in reporting. For further information on Nature Portfolio policies, see our Editorial Policies and the Editorial Policy Checklist.

Statistics

For all statistical analyses, confirm that the following items are present in the figure legend, table legend, main text, or Methods section.

n/a	Confirmed	
☐	☒	The exact sample size (*n*) for each experimental group/condition, given as a discrete number and unit of measurement
☐	☒	A statement on whether measurements were taken from distinct samples or whether the same sample was measured repeatedly
☐	☒	The statistical test(s) used AND whether they are one- or two-sided<br>*Only common tests should be described solely by name; describe more complex techniques in the Methods section.*
☒	☐	A description of all covariates tested
☐	☒	A description of any assumptions or corrections, such as tests of normality and adjustment for multiple comparisons
☐	☒	A full description of the statistical parameters including central tendency (e.g. means) or other basic estimates (e.g. regression coefficient) AND variation (e.g. standard deviation) or associated estimates of uncertainty (e.g. confidence intervals)
☐	☒	For null hypothesis testing, the test statistic (e.g. *F*, *t*, *r*) with confidence intervals, effect sizes, degrees of freedom and *P* value noted<br>*Give P values as exact values whenever suitable.*
☒	☐	For Bayesian analysis, information on the choice of priors and Markov chain Monte Carlo settings
☐	☒	For hierarchical and complex designs, identification of the appropriate level for tests and full reporting of outcomes
☒	☐	Estimates of effect sizes (e.g. Cohen's *d*, Pearson's *r*), indicating how they were calculated

Our web collection on statistics for biologists contains articles on many of the points above.

Software and code

Policy information about availability of computer code

Data collection	BD FACSDiva Software v9.2 is used for flow cytometry.
Data analysis	For flow cytometry: FlowJo V.10.8.1 software; For data analysis: Graphpad Prism 10.

For manuscripts utilizing custom algorithms or software that are central to the research but not yet described in published literature, software must be made available to editors and reviewers. We strongly encourage code deposition in a community repository (e.g. GitHub). See the Nature Portfolio guidelines for submitting code & software for further information.

Data

Policy information about availability of data

All manuscripts must include a data availability statement. This statement should provide the following information, where applicable:

- Accession codes, unique identifiers, or web links for publicly available datasets
- A description of any restrictions on data availability
- For clinical datasets or third party data, please ensure that the statement adheres to our policy

The data that support the findings of this study are available from the corresponding author upon reasonable request.

Research involving human participants, their data, or biological material

Policy information about studies with human participants or human data. See also policy information about sex, gender (identity/presentation), and sexual orientation and race, ethnicity and racism.

Reporting on sex and gender	Sex was determined based on biological attributes documented in medical records, and no gender-related self-reporting or social identity assessments were involved. All participants provided informed consent, including consent for the collection and analysis of individual-level clinical data. However, sex-based or gender-based subgroup analyses were not performed, as the study did not involve bio-marker stratification or multivariate analyses based on sex or gender. Disaggregated sex data are provided in the Source Data file.
Reporting on race, ethnicity, or other socially relevant groupings	Race, ethnicity, or other socially relevant groupings were not considered in the study design.
Population characteristics	The key inclusion criteria were as follows: age ≥ 18 years old; ER+ and/or progesterone receptor-positive (PR+) tumors (≥ 10%) and HER2- local recurrent or metastatic disease diagnosed by pathohistological or cytological examination; at least one extracranial measured lesion per Response Evaluation Criteria in Solid Tumors (RECIST, version 1.1); received ≤ 1 line of chemotherapy for recurrent or metastatic BC; experienced disease recurrence and/or metastasis after treatment with a CDK4/6i (palbociclib, abemaciclib, or ribociclib); received ≤ 3 lines of ET for recurrent or metastatic BC; ECOG performance status of 0–2; life expectancy of ≥ 6 months; normal hematologic, cardiac, hepatic, renal, and thyroid function. Patients previously received HDACi therapy; prior treatment with dalpiciclib; MRI or lumbar puncture-confirmed leptomeningeal metastasis, or imaging-confirmed central nervous system metastasis were excluded from this study. A total of 22 patients were enrolled in this study, with a median age of 51.5 years (range: 33–63). Most patients (95.5%, 21/22) had an ECOG performance status of 0, while only one patient (4.5%) had an ECOG score of 1. In terms of hormone receptor (HR) status, 59.1% (13/22) of patients were ER+/PR+, and 40.9% (9/22) were ER+/PR−. The majority of patients (59.1%, 13/22) had ≥ 3 metastatic sites, with visceral metastases (77.3%, 17/22) and bone metastases (72.7%, 16/22) being the most common. Lymph node metastases were present in 59.1% (13/22) of patients. Regarding prior treatments, 81.8% (18/22) of patients had received chemotherapy for recurrent/metastatic disease, and 54.5% (12/22) had received ≥ 3 lines of prior therapy. Among CDK4/6i in prior treatment, abemaciclib was the most frequently used (81.8%, 18/22), followed by palbociclib (40.9%, 9/22) and ribociclib (4.5%, 1/22). The majority of patients (72.7%, 16/22) had received CDK4/6i for more than 12 months.
Recruitment	The participants were recruited according to the protocol which was approved by Ethics Committee of Hainan Jialong Internet Hospital. All the participants signed the Informed Consent Form.
Ethics oversight	The study was conducted in compliance with the Declaration of Helsinki and Good Clinical Practice (GCP) guidelines. The protocol and its amendments approved by Ethics Committee. Informed consent was obtained from each patient prior to treatment.

Note that full information on the approval of the study protocol must also be provided in the manuscript.

Field-specific reporting

Please select the one below that is the best fit for your research. If you are not sure, read the appropriate sections before making your selection.

☒ Life sciences ☐ Behavioural & social sciences ☐ Ecological, evolutionary & environmental sciences

For a reference copy of the document with all sections, see nature.com/documents/nr-reporting-summary-flat.pdf

Life sciences study design

All studies must disclose on these points even when the disclosure is negative.

Sample size	This is a single-arm clinical trial utilized BOIN design to determine the MTD. The target dose-limiting toxicity (DLT) rate (defined as the number of patients experiencing DLT at the current dose /the total number of evaluable patients treated at the same dose) is set at 0.33. The first treatment cycle was used as the DLT observation window to determine dose escalation or de-escalation. Each dose group could enroll up to 12 patients, with a maximum of 30 patients for the entire study.
Data exclusions	Nodata were excluded from the analysis
Replication	The findings of this trial are not replicated or reproduced because there was not enough material.
Randomization	N/A
Blinding	N/A

Reporting for specific materials, systems and methods

We require information from authors about some types of materials, experimental systems and methods used in many studies. Here, indicate whether each material, system or method listed is relevant to your study. If you are not sure if a list item applies to your research, read the appropriate section before selecting a response.

Materials & experimental systems

n/a	Involved in the study	
☐	☒	Antibodies
☐	☒	Eukaryotic cell lines
☒	☐	Palaeontology and archaeology
☒	☐	Animals and other organisms
☐	☒	Clinical data
☒	☐	Dual use research of concern
☒	☐	Plants

Methods

n/a	Involved in the study	
☒	☐	ChIP-seq
☐	☒	Flow cytometry
☒	☐	MRI-based neuroimaging

Antibodies

Antibodies used	Phosphor-retinoblastoma (S807) (pRb) antibody (Abmart, T55499S, 1:500), retinoblastoma (Rb) antibody (Abmart, T55661S, 1:1000), cyclin E2 antibody (Selleck, F2373, 1:1000), cyclin D1 antibody (Selleck, F0137, 1:1000), CDK4 antibody (Selleck, F0322, 1:1000), CDK2 antibody (Selleck, F0022, 1:1000), Bax antibody (Selleck, F0037, 1:1000), p21 antibody(Selleck, F0022, 1:1000), β-actin antibody (Abmart, P30002, 1:2000), and HRP-conjugated secondary mouse & rabbit antibody (Abmart, M21003, 1:1000).
Validation	For western blot, all antibodies were used as validated by the manufacturer for their specific assay according to their data sheet. In addition, the blotting bands were consistent with the predicted molecular weight of the protein. Abmart and Selleck: antibodies were validated by the manufacturer and independently for specificity within our lab group by western blot.

Eukaryotic cell lines

Policy information about cell lines and Sex and Gender in Research

Cell line source(s)	Human breast cancer cell line ZR-75-1 and MCF7 are obtained from ATCC and cultured according to standard protocols.
Authentication	The cell lines used in manuscript were not contained in the list of misidentified cell lines. The cell lines used were not laboratory authenticated after purchase.
Mycoplasma contamination	All cell lines used tested negative for mycoplasma contamination.
Commonly misidentified lines (See ICLAC register)	o commonly misidentified cell lines from the ICLAC Register were used in the study.

Clinical data

Policy information about clinical studies

All manuscripts should comply with the ICMJE guidelines for publication of clinical research and a completed CONSORT checklist must be included with all submissions.

Clinical trial registration	NCT05586841
Study protocol	The study protocol has been submitted with the manuscript.
Data collection	Between January 2023 and January 2025, data were collected from 22 patients with HR+/HER2− breast cancer who had experienced disease progression following CDK4/6 inhibitor treatment at the Fifth Medical Center of the Chinese PLA General Hospital.
Outcomes	The primary endpoint was maximum tolerated dose (MTD), and the secondary endpoints were objective response rate (ORR), progression-free survival (PFS), disease control rate (DCR) and safety analysis. Eligible patients underwent weekly (±2 days) routine blood tests, as well as liver and renal function assessments after drug administration. Tumor response was evaluated every two cycles (±7 days) using imaging based on RECIST 1.1 criteria, until disease progression or the initiation of a new anti-tumor therapy. Patients who experienced disease progression or started a new treatment were followed up every 12 weeks to assess survival status. The assessment of safety included adverse events (AE), clinical laboratory examinations, vital signs, and physical examinations. The AEs were documented and graded according to the NCI Common Terminology Criteria for Adverse Events (version 5.0). Furthermore, we also explored the potential biomarkers associated with outcomes

Plants

Seed stocks: N/A

Novel plant genotypes: N/A

Authentication: N/A

Flow Cytometry

Plots

Confirm that:

- [x] The axis labels state the marker and fluorochrome used (e.g. CD4-FITC).
- [x] The axis scales are clearly visible. Include numbers along axes only for bottom left plot of group (a 'group' is an analysis of identical markers).
- [x] All plots are contour plots with outliers or pseudocolor plots.
- [x] A numerical value for number of cells or percentage (with statistics) is provided.

Methodology

Sample preparation	Cell cycle analysis was performed according to the instructions of the Cell Cycle Detection Kit (Biosharp, BL114A). Harvested cells were fixed and incubated in working solution. The stained cells were centrifuged and re-suspended in PBS for flow cytometry (FCM) analysis.
Instrument	Samples were measured on a Fortessa (BD Biosciences, Heodelberg, Germany)
Software	BD FACSDiva Software v9.2
Cell population abundance	These cells were not sorted.
Gating strategy	The cells were identified by their FSC and SSC profiles. A polygon gate was drawn on a FSC-A vs SSC-A dot plot to include the cell population. The boundaries between positive and negative gates were set based upon an unstained control.

- [x] Tick this box to confirm that a figure exemplifying the gating strategy is provided in the Supplementary Information.
