# Supplementary material for: Dalpiciclib plus chidamide in HR + /HER2−advanced breast cancer after CDK4/6 inhibitor failure: a phase Ib trial
Source: Nat Commun. 2026 Mar 20;17:5177. doi: 10.1038/s41467-026-70650-6 (PMC13254248; doi:10.1038/s41467-026-70650-6)
Supplement: Supplementary file 3 — Transparent Peer Review file [file 41467_2026_70650_MOESM3_ESM.pdf]

nature portfolio

Peer Review File

Dalpiciclib Plus Chidamide in HR+/HER2−Advanced Breast Cancer After CDK4/6 Inhibitor Failure: A Phase Ib Trial

Corresponding Author: Professor Tao Wang

This file contains all reviewer reports in order by version, followed by all author rebuttals in order by version.

Version 0:

Reviewer comments:

Reviewer #1

(Remarks to the Author)
Zhou and colleagues present a phase 1B trial exploring the combination of the CDK4/6 inhibitor dalpiciclib with the HDAC inhibitor chidamide for patients with HR positive, HER2 negative advanced breast cancer. This single arm study employed a dose escalation design with several preselected doses of each agent based upon prior experiences. 22 patients were enrolled, and there were a significant number of toxicity events noted across the entire cohort, predominantly cytopenias. The authors defined a maximum tolerated dose combination. The objective response rate ranged from 9 to 17% depending on the cohort. The overall median progression free survival was approximately 6 months, though notably over 12 months at the MTD. The degree of clinical benefit appears to be shorter in patients with PIK3CA mutations. The authors also provide some in vitro data demonstrating potential synergy between a combination.

There are a number of limitations related to this study. Though novel combinations to address resistance to CDK inhibitor therapy represents an important area of ongoing research, the sample size here is small and the rationale behind dosing and MTD assessment is not entirely clear. The safety data is not adequately presented in the manuscript, and it is difficult to draw large conclusions from the efficacy data given very small subsets (for example restricting below 22 patients to assess PFS in the MTD). It is not clear whether this is a true signal or perhaps an impact of the small sample size. There are a number of adjustments which could strengthen the manuscript:

- Phase 1 trials with novel combinations should primarily be focused upon safety assessments. There is no detailed safety table highlighting the various toxicities experienced at each dose level, though there is some of this detail provided in the text. Much more insight into the toxicity experience across the entire cohort needs to be provided, including in either a table and/or figure. This information should precede any visualization or discussion of clinical activity in this very small safety cohort. It is also not entirely clear how the authors established an MTD at a combination where 100% of the patients required dose reductions in Chidamide. Further explanation of this rationale should be provided.

- The biomarker analysis is interesting, though again somewhat limited by sample size with some counterintuitive findings. For example, only 18% of patients demonstrated an ESR 1 mutation despite fairly high degrees of pretreatment including with prior antiestrogen therapy and CDK blockade. One might expect this number to be higher based upon contemporary experiences. The authors also note that 36.4% of patients had "unknown genetic alterations." The meaning of this is not clear, and should be explained in more detail.

- The in vitro combination data is helpful to place the rationale behind the study and context. It may be prudent to move this material to the beginning of the paper, to set the stage for subsequent efforts in clinic to combine these agents.

- The median PFS of greater than 1 year in group C is notable, and would be among the best clinical performances in the post CDK setting. Whether this is a realistic assessment is not clear given the very small sample size and the overall median PFS of approximately 6 months, which is more typical in this setting. The authors should provide more insight into the median PFS of other dosing subgroups outside of group C. It is possible that some of these differences are simply related to small sample size, and this should be described as a very important limitation to interpreting any clinical data here. The

authors also suggest EMBER3 represents the longest PFS in previously published second line studies, though this is not entirely true, as the phase 2 ELAINE2 study incorporating lasofoxifene and abemaciclib had a median PFS exceeding 12 months, and this should be mentioned in the discussion.

- In the discussion, the authors comment that ESR 1 mutations confer reduced sensitivity to CDK 4/6 inhibitors, this is not necessarily true. They should emphasize that this is a matter of debate, and many publications have demonstrated ESR1 mutations occurring in tumors that are sensitive to CDK blockade. There is also prior laboratory data supporting this finding (of CDK4/6i activity in ESR1m models).

- There are number of grammatical mistakes throughout the manuscript, the entire paper needs to be carefully edited and corrected.

Reviewer #2

(Remarks to the Author)
This study is a Phase Ib trial aimed at determining the maximum tolerated dose (MTD) of combination therapy with dalpiciclib and chidamide for HR+/HER2− advanced breast cancer after failure of a CDK4/6 inhibitor. The study adopts the Bayesian Optimal Interval (BOIN) design, and Group C (dalpiciclib 100 mg/d, 325 25 mg chidamide BIW) was determined to be the MTD. Overall, I think the manuscript is well written; below are comments mainly from a statistical perspective.

1. The rationale for why Group C was selected as the MTD is somewhat unclear. I recommend adding a figure that shows the dose-escalation/de-escalation path similar to Figure 2 in Yuan Y et al. (Clin Cancer Res 2016) and then explaining why Group C was determined to be the MTD.
2. In addition to the DLT incidence proportion in Group C, please also present its 95% confidence interval.
3. I could not find a statement that the cohort size is 3; I think it would be better to include this.
4. In the Efficacy section, comparisons of PD rates across groups are shown, but from a statistical standpoint it is difficult to evaluate “higher” or “lower.”
5. Some abbreviations defined in the Methods are used earlier in the text. Please review and correct this.

Version 1:

Reviewer comments:

Reviewer #1

(Remarks to the Author)
Thank you for addressing my prior comments

Reviewer #2

(Remarks to the Author)
I have no further comment.

Open Access This Peer Review File is licensed under a Creative Commons Attribution 4.0 International License, which permits use, sharing, adaptation, distribution and reproduction in any medium or format, as long as you give appropriate credit to the original author(s) and the source, provide a link to the Creative Commons license, and indicate if changes were made.
In cases where reviewers are anonymous, credit should be given to 'Anonymous Referee' and the source.
The images or other third party material in this Peer Review File are included in the article's Creative Commons license, unless indicated otherwise in a credit line to the material. If material is not included in the article's Creative Commons license and your intended use is not permitted by statutory regulation or exceeds the permitted use, you will need to obtain permission directly from the copyright holder.
To view a copy of this license, visit https://creativecommons.org/licenses/by/4.0/

Reviewer #1 (Remarks to the Author); expert in breast cancer:

Zhou and colleagues present a phase 1B trial exploring the combination of the CDK4/6 inhibitor dalpiciclib with the HDAC inhibitor chidamide for patients with HR positive, HER2 negative advanced breast cancer. This single arm study employed a dose escalation design with several preselected doses of each agent based upon prior experiences. 22 patients were enrolled, and there were a significant number of toxicity events noted across the entire cohort, predominantly cytopenias. The authors defined a maximum tolerated dose combination. The objective response rate ranged from 9 to 17% depending on the cohort. The overall median progression free survival was approximately 6 months, though notably over 12 months at the MTD. The degree of clinical benefit appears to be shorter in patients with PIK3CA mutations. The authors also provide some in vitro data demonstrating potential synergy between a combination.

There are a number of limitations related to this study. Though novel combinations to address resistance to CDK inhibitor therapy represents an important area of ongoing research, the sample size here is small and the rationale behind dosing and MTD assessment is not entirely clear. The safety data is not adequately presented in the manuscript, and it is difficult to draw large conclusions from the efficacy data given very small subsets (for example restricting below 22 patients to assess PFS in the MTD). It is not clear whether this is a true signal or perhaps an impact of the small sample size.

Response: Thank you for your thoughtful comments. We agree that this study has inherent limitations, particularly related to the small sample size and the exploratory nature of the efficacy analyses. As a phase I trial, the primary objective was to evaluate safety and determine the maximum tolerated dose (MTD); therefore, efficacy outcomes were considered exploratory and not powered for definitive conclusions. This naturally limits the generalizability of the efficacy findings.

Regarding your specific comments, we address each point individually in the responses below.

There are a number of adjustments which could strengthen the manuscript:

- Phase 1 trials with novel combinations should primarily be focused upon safety assessments. There is no detailed safety table highlighting the various toxicities experienced at each dose level, though there is some of this detail provided in the text. Much more insight into the toxicity experience across the entire cohort needs to be provided, including in either a table and/or figure. This information should precede any visualization or discussion of clinical activity in this very small safety cohort. It is also not entirely clear how the authors established an MTD at a combination where 100% of the patients required dose reductions in Chidamide. Further explanation of this rationale should be provided.

Response: We appreciate the reviewer's emphasis on the importance of safety

assessment. In accordance with this, we have revised the Dose Escalation section to provide a detailed narrative description of the dose-limiting toxicities (DLTs) observed across all four dose cohorts, presented before the clinical activity data.

As specified in the study protocol, the DLT observation window was defined as the first treatment cycle and served as the basis for dose-escalation and de-escalation decisions under the Bayesian Optimal Interval (BOIN) design. DLTs were observed in cohort A (1/4, 25%) and cohort C (2/12, 16.7%), all of which were grade 4 thrombocytopenia. No DLTs occurred in cohorts B or D. These findings are now clearly summarized in Table 2.

According to the BOIN design, the first cohort (Group D) received dalpiciclib 100 mg once daily (QD) plus chidamide 20 mg twice weekly (BIW). None of the initial three evaluable patients experienced a DLT (observed toxicity rate < 0.26), so the dose was escalated to dalpiciclib 125 mg/day plus chidamide 20 mg BIW (Group B). Again, no DLTs occurred (observed toxicity rate < 0.26), allowing escalation to dalpiciclib 100 mg/day plus chidamide 25 mg BIW (Group C). In Group C, four evaluable patients were enrolled and one patient experienced a DLT (25%), permitting escalation to dalpiciclib 125 mg/day plus chidamide 25 mg BIW (Group A), according to the BOIN design (observed toxicity rate < 0.26). In Group A, 1 of 4 (25%) evaluable patients experienced a DLT.

According to the BOIN design, the observed DLT rate in Group A (25%) was below the escalation threshold, which would have permitted continued enrollment (Figure S1). However, all four patients in this cohort experienced treatment interruptions due to adverse events during the first two cycles, reflecting poor tolerability at this dose level. In contrast, in Group C, acceptable safety and tolerability were observed, and two patients achieved partial responses at the first efficacy assessment. Taking both safety and preliminary efficacy into consideration, the dose was de-escalated to Group C for further evaluation. Group C required no further de-escalation and ultimately enrolled 12 patients, reaching the protocol-defined completion criteria. Although all patients in Group C eventually required chidamide dose reductions, all of these occurred beyond the first treatment cycle and therefore did not meet the protocol definition of DLT. Based on these findings, Group C was determined to represent the MTD. The full safety data by dose level are summarized in Table S3. (**Line 192-236**)

- The biomarker analysis is interesting, though again somewhat limited by sample size with some counterintuitive findings. For example, only 18% of patients demonstrated an ESR 1 mutation despite fairly high degrees of pretreatment including with prior antiestrogen therapy and CDK blockade. One might expect this number to be higher based upon contemporary experiences. The authors also note that 36.4% of patients had "unknown genetic alterations." The meaning of this is not clear, and should be explained in more detail.

Response: We thank the reviewer for the valuable comment. Among the 22 patients enrolled, genetic analysis was conducted in 14 patients. Within this subset, *ESR1* mutations were detected in 4 patients, resulting in a mutation rate of 28.6% (4/14), which aligns with previously reported frequencies in heavily pretreated HR+/HER2−

breast cancer populations. The previously reported figure of 18.2% reflected the proportion of *ESR1* mutations in the entire study cohort (4/22), which included patients without available genomic data. Furthermore, we have updated Table 1 and the corresponding sections of the manuscript. (**Line 290-295**)

- The in vitro combination data is helpful to place the rationale behind the study and context. It may be prudent to move this material to the beginning of the paper, to set the stage for subsequent efforts in clinic to combine these agents.

Response: We thank the reviewer for this valuable suggestion. In response, we have revised the structure of the Results section to present the *in vitro* combination data at the beginning, prior to the clinical findings. This change better supports the translational rationale of the study and aligns the scientific logic from preclinical hypothesis to clinical trial. (**Line 93-131**)

- The median PFS of greater than 1 year in group C is notable, and would be among the best clinical performances in the post CDK setting. Whether this is a realistic assessment is not clear given the very small sample size and the overall median PFS of approximately 6 months, which is more typical in this setting. The authors should provide more insight into the median PFS of other dosing subgroups outside of group C. It is possible that some of these differences are simply related to small sample size, and this should be described as a very important limitation to interpreting any clinical data here. The authors also suggest EMBER3 represents the longest PFS in previously published second line studies, though this is not entirely true, as the phase 2 ELAINE2 study incorporating lasofoxifene and abemaciclib had a median PFS exceeding 12 months, and this should be mentioned in the discussion.

Response: We thank the reviewer for this insightful and constructive comment. We fully acknowledge that the median PFS of 12.29 months observed in cohort C is derived from a small number of patients (n = 12), and should be interpreted with caution given the exploratory nature of this phase Ib study. The limited sample size, especially in the other dose cohorts (n = 3–4 per group), precluded reliable estimation of median PFS in those subgroups. However, for reference, the observed PFS ranges in cohorts A, B, and D were 1.97–9.99 months, 2.17–7.10 months, and 5.78–6.64 months, respectively. These observations are also illustrated in the swimmer plot (Figure 3), which depicts treatment duration and best overall response for individual patients across the different dose cohorts. To further evaluate the promising activity observed in cohort C, a phase II expansion study (NCT06556862) is currently underway to validate these findings in a larger patient population. Additionally, we appreciate the reviewer's note on the ELAINE2 study. We have now included this reference in the Discussion, and acknowledge that the phase II ELAINE2 trial (lasofoxifene plus abemaciclib) also demonstrated a median PFS exceeding 12 months in a similar post-CDK4/6i setting. (**Line 424-427**)

- In the discussion, the authors comment that ESR 1 mutations confer reduced sensitivity to CDK 4/6 inhibitors, this is not necessarily true. They should emphasize

that this is a matter of debate, and many publications have demonstrated ESR1 mutations occurring in tumors that are sensitive to CDK blockade. There is also prior laboratory data supporting this finding (of CDK4/6i activity in ESR1m models).

Response: We thank the reviewer for this important clarification. We agree that the relationship between *ESR1* mutations and CDK4/6i sensitivity remains an area of ongoing investigation and debate. We have revised the Discussion section to reflect this complexity and acknowledge the conflicting evidence in the literature. (**Line 481-490**)

- There are number of grammatical mistakes throughout the manuscript, the entire paper needs to be carefully edited and corrected.

Response: Thank you for your comment. The manuscript has been fully revised for grammar and language.

Reviewer #2 (Remarks to the Author); expert in statistics and clinical trial design:

This study is a Phase Ib trial aimed at determining the maximum tolerated dose (MTD) of combination therapy with dalpiciclib and chidamide for HR+/HER2− advanced breast cancer after failure of a CDK4/6 inhibitor. The study adopts the Bayesian Optimal Interval (BOIN) design, and Group C (dalpiciclib 100 mg/d, 325 25 mg chidamide BIW) was determined to be the MTD. Overall, I think the manuscript is well written; below are comments mainly from a statistical perspective.

1. The rationale for why Group C was selected as the MTD is somewhat unclear. I recommend adding a figure that shows the dose-escalation/de-escalation path similar to Figure 2 in Yuan Y et al. (Clin Cancer Res 2016) and then explaining why Group C was determined to be the MTD.

Response: Thank you for your valuable comment. Dose escalation in this study was guided by a predefined Bayesian Optimal Interval (BOIN) design, based on the cumulative number of subjects who experienced a DLT at the current dose level, with DLTs defined in the protocol and SAP submitted with the manuscript. Acceptable safety and tolerability were assessed during the first treatment cycle.

The first cohort (Group D) received dalpiciclib 100 mg once daily (QD) plus chidamide 20 mg twice weekly (BIW). None of the initial three evaluable patients experienced a DLT (observed toxicity rate < 0.26), so the dose was escalated to dalpiciclib 125 mg/day plus chidamide 20 mg BIW (Group B). Again, no DLTs occurred (observed toxicity rate < 0.26), allowing escalation to dalpiciclib 100 mg/day plus chidamide 25 mg BIW (Group C). In Group C, four evaluable patients were enrolled and one patient experienced a DLT (25%), permitting escalation to dalpiciclib 125 mg/day plus chidamide 25 mg BIW (Group A), according to the BOIN design (observed toxicity rate < 0.26). In Group A, 1 of 4 (25%) evaluable patients experienced a DLT.

According to the BOIN design, the observed DLT rate in Group A (25%) was below the escalation threshold, which would have permitted continued enrollment (Figure S1). However, all four patients in this cohort experienced treatment interruptions due to adverse events during the first two cycles, reflecting poor tolerability at this dose level.

In contrast, in Group C, acceptable safety and tolerability were observed, and two patients achieved partial responses at the first efficacy assessment. Taking both safety and preliminary efficacy into consideration, the dose was de-escalated to Group C for further evaluation.

Under the predefined BOIN decision rules, Group C required no further de-escalation and ultimately enrolled 12 patients, reaching the protocol-defined completion criteria. Although all patients in Group C eventually required chidamide dose reductions, all of these occurred beyond the first treatment cycle and therefore did not meet the protocol definition of DLT. Based on these findings, Group C was determined to represent the MTD. (**Line 192-236**)

2. In addition to the DLT incidence proportion in Group C, please also present its 95% confidence interval.

Response: Thank you for your comment. The 95% confidence intervals for DLT rates in each cohort were calculated based on the Clopper-Pearson method. Due to the small sample sizes and wide intervals, these values were not presented in the main text but are available upon request (Table 2). (**Line 201-204**)

3. I could not find a statement that the cohort size is 3; I think it would be better to include this.

Response: Thank you for your suggestion. We have added a statement to clarify the cohort sizes. (**Line 136-138**)

4. In the Efficacy section, comparisons of PD rates across groups are shown, but from a statistical standpoint it is difficult to evaluate "higher" or "lower."

Response: We thank the reviewer for this kindly comment. The description has been revised according to your suggestion. (**Line 280-281**)

5. Some abbreviations defined in the Methods are used earlier in the text. Please review and correct this.

Response: Thanks for the valuable comment. We have reviewed the manuscript and corrected the abbreviations.

REVIEWERS' COMMENTS

Reviewer #1 (Remarks to the Author):

Thank you for addressing my prior comments

Response: Thank you for your positive feedback and for recognizing our revisions; we greatly appreciate your support.

Reviewer #2 (Remarks to the Author):

I have no further comment.

Response: Thank you for your positive feedback and for recognizing our revisions; we greatly appreciate your support.
